# A Novel Analog of the Natural Product Fraxinellone Protects against Endogenous and Exogenous Neurotoxicants

**DOI:** 10.1021/acschemneuro.4c00090

**Published:** 2024-06-26

**Authors:** Anna E. Bartman, Mersad Raeisi, Clarence D. Peiris, Isabella E. Jacobsen, David B.C. Martin, Jonathan A. Doorn

**Affiliations:** † Department of Pharmaceutical Sciences & Experimental Therapeutics, College of Pharmacy, University of Iowa, Iowa City, Iowa 52242, United States; ‡ Department of Chemistry, College of Liberal Arts & Sciences, University of Iowa, Iowa City, Iowa 52242, United States

**Keywords:** glutamate, fraxinellone, Nrf2-Keap1, reactive oxygen species, excitotoxicity

## Abstract

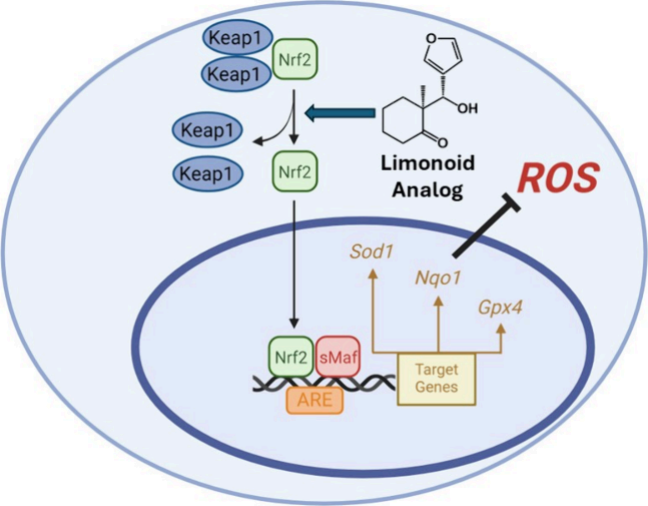

Numerous insults, both endogenous (e.g., glutamate) and exogenous (e.g., pesticides), compromise the function of the nervous system and pose risk factors for damage or later disease. In previous reports, limonoids such as fraxinellone showed significant neuroprotective activity against glutamate (Glu) excitotoxicity and reactive oxygen species (ROS) production in vitro, albeit with minimal mechanistic information provided. Given these findings, a library of novel fraxinellone analogs (including analogs 1 and 2 described here) was synthesized with the goal of identifying compounds exhibiting neuroprotection against insults. Analog 2 was found to be protective against Glu-mediated excitotoxicity with a measured EC_50_ of 44 and 39 nM for in vitro assays using PC12 and SH-SY5Y cells, respectively. Pretreatment with analog 2 yielded rapid induction of antioxidant genes, namely, *Gpx4*, *Sod1*, and *Nqo1*, as measured via qPCR. Analog 2 mitigated Glu-mediated ROS. Cytoprotection could be replicated using sulforaphane (SFN), a Nrf2 activator, and inhibited via ML-385, which inhibits Nrf2 binding to regulatory DNA sequences, thereby blocking downstream gene expression. Nrf2 DNA-binding activity was demonstrated using a Nrf2 ELISA-based transcription factor assay. In addition, we found that pretreatment with the thiol N-acetyl Cys completely mitigated SFN-mediated induction of antioxidant genes but had no effect on the activity of analog 2, suggesting thiol modification is not critical for its mechanism of action. In summary, our data demonstrate a fraxinellone analog to be a novel, potent, and rapid activator of the Nrf2-mediated antioxidant defense system, providing robust protection against insults.

## Introduction

Numerous insults, both endogenous (e.g., Glu) and exogenous (e.g., pesticides), compromise the function of the nervous system by producing an imbalance in ROS and thereby serve as risk factors for cell injury and later disease.^1−3^ In particular, extracellular levels of the excitatory neurotransmitter glutamate (Glu) increase in response to injury, such as stroke, as well as several diseases, including amyotrophic lateral sclerosis (ALS), epilepsy, and Alzheimer’s disease.^4−6^ Additionally, aberrant Glu levels and signaling represent a mechanistic component of damage to the nervous system following exposure to chemical threats such as organophosphate nerve agents.^7^ The aberrant synaptic signaling via Glu leads to what is referred to as excitotoxicity, producing excessive levels of ROS and several other damaging downstream mediators (e.g., inflammatory).^8,9^ In addition to Glu, numerous environmental insults, such as pesticides, are also known to induce oxidative stress and cause injury to neurons.^10^

**Scheme 1:**
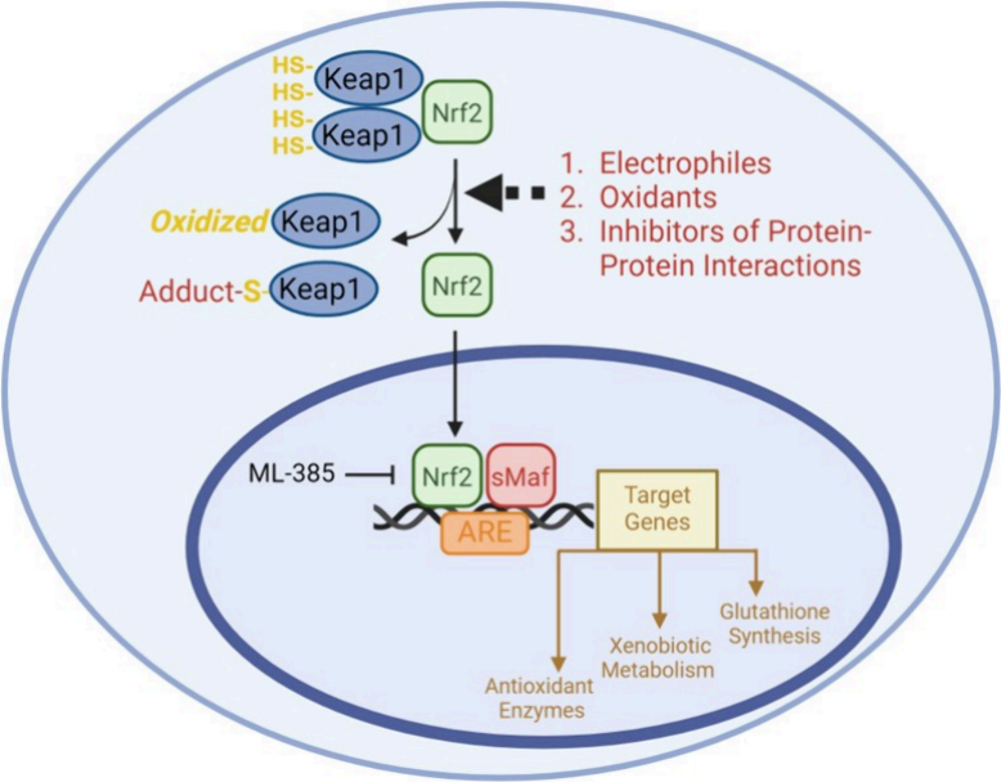
Nrf2/Keap1 Signaling Mediates Antioxidant Response Activation and release of Nrf2 occurs via the: (1) reaction of an electrophile with Keap1 thiols, such as SFN; (2) oxidation of Keap1 thiols via ROS; or (3) noncovalent disruption of the Keap1-Nrf2 interaction. Upon dissociation from Keap1, Nrf2 translocates to the nucleus and binds with sMaf to the antioxidant response element (ARE), yielding expression of genes involved in xenobiotic metabolism, ROS mitigation, and GSH synthesis. The interaction of Nrf2 with sMaf and the ARE can be inhibited by ML-385. Figure created with BioRender.com.

An effective cellular mechanism of defense against elevated oxidative stress is through activation of the Kelch-like ECH-associated protein 1 (Keap1), nuclear factor erythroid 2-related factor 2 (Nrf2), and antioxidant response elements (ARE) signaling pathway (Scheme 1).^11,12^ Under normal conditions, Keap1 exists as a homodimer and forms a complex with Nrf2 with two different affinity binding sites on the Neh2 domain of Nrf2 (ETGE motif and DLG motif). The ETGE motif has stronger binding to Keap1 compared to the DLG motif and is responsible for the recruitment of Nrf2 by Keap1. The DLG motif of the Neh2 domain is responsible for locking the correct position for ubiquitin signaling for Nrf2 degradation. While complexed together, Keap1 suppresses the activity of Nrf2 by associating with a functional E3 ubiquitin ligase complex by the scaffold protein Cullin3 (Cul3). Cul3 then facilitates Rbx1-mediated polyubiquitination of Nrf2, thus resulting in the degradation of Nrf2 via 26S proteasome.^12,13^

Following the production of excessive ROS, proximal/adjacent Cys residues of Keap1 are oxidized to disulfides, thus resulting in the release of Nrf2.^14^ More specifically, oxidative stress prevents Keap1-Nrf2-Cul3 assembly and thus, Nrf2 ubiquitination.^15,16^ In addition, disruption of the complex can occur by covalent modification of the Cys residues, such as sulforaphane (SFN) as an example, or via noncovalent inhibitors targeting the Nrf2-Keap1 interface.^17,18^ Upon release, Nrf2 first accumulates in the cytosol before translocating into the nucleus, where it forms a heterodimer with small musculoaponeurotic fibrosarcoma (sMaf) proteins and binds ARE. Subsequently, this modulates the transcription of a vast array of antioxidant genes as a defense against elevated ROS as well as inhibiting inflammatory events given cross-talk with NF-kB signaling.^9,19,20^ Due to the vital role of Nrf2 in antioxidant defense against endogenous and exogenous insults, Nrf2-targeting agents have been investigated extensively over the last several years for the potential to mitigate diseases with oxidative stress and inflammation underlying pathology.^16,21^ Several Nrf2 activators are being marketed to treat conditions such as multiple sclerosis and psoriasis, and, in various stages of development (preclinical to Phase III), to address other disease states such as ALS, subarachnoid hemorrhage, and Friedreich Ataxia.^21,22^ In addition, Nrf2 activators may have promise to mitigate damage from chemical threats, such as organophosphate nerve agents, prophylactically or post-treatment.^23,24^

**Figure 1:**
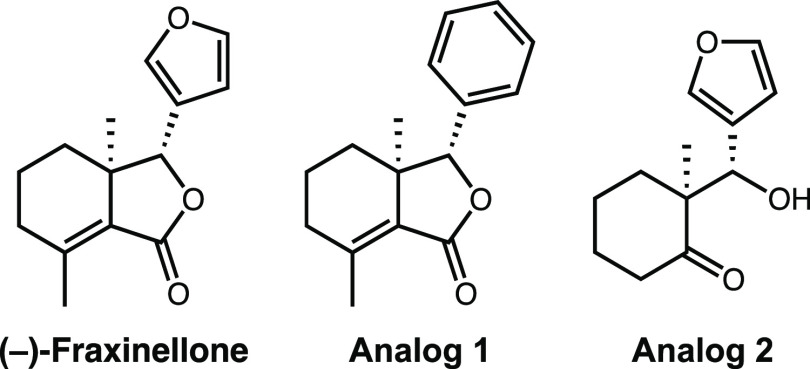
Structures of fraxinellone and analogs.

In previous reports, limonoids isolated from *Dictamnus dasycarpus*, such as fraxinellone, showed notable neuroprotective activity against Glu excitotoxicity and oxidative stress in both primary cultured rat cortical cells and astrocytes, although no mechanism was proposed.^25−27^ Given these results, a primary goal of this study was to synthesize new fraxinellone analogs with simpler structure that were better able to protect against endogenous and exogenous toxicants, when compared to fraxinellone and other limonoid natural products (Figure 1).^25,28^ Each analog tests the importance of the fraxinellone structure, including the furan ring (analog 1) and the bicyclic core (Analog 2). A second goal of this work was to identify the mechanism of action for the analogs. In vitro methods were used to assess the protective activity of the novel fraxinellone analogs against Glu excitotoxicity and whether these analogs mitigated oxidative stress. Surprisingly, our studies revealed that one of the fraxinellone analogs effectively mitigated oxidative stress at low concentrations through rapid and potent activation of the Nrf2/Keap1 pathway via a potentially novel mechanism compared to known activators, such as SFN.^29^ Our results indicate a novel mechanism of action compared to known Nrf2 activators, which are electrophiles or oxidants that rely on thiol modification.^30^ The fraxinellone analogs are resistant to thiol reactivity, thereby conferring stability and minimizing off-target reactions as improved characteristics to be considered for therapeutic development.

## Results and Discussion

### Synthesis of Fraxinellone and Analogs

**Scheme 2:**
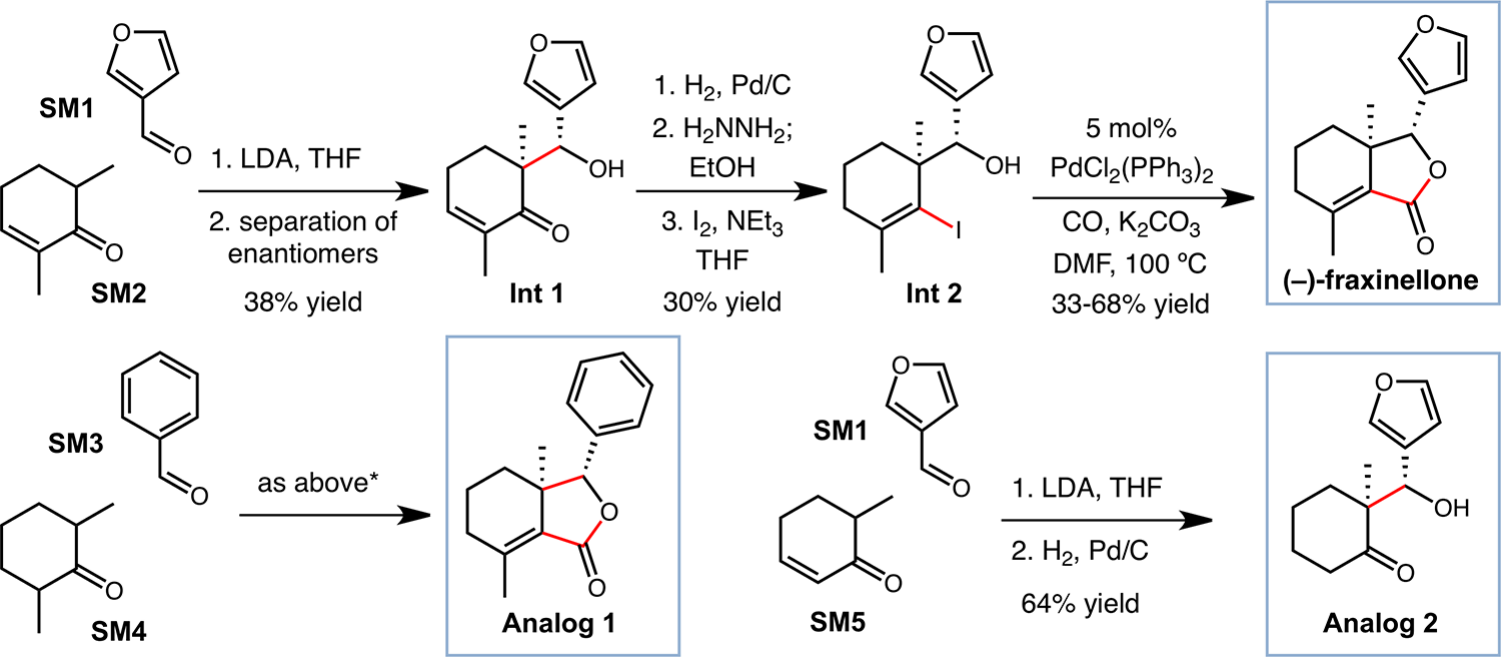
Synthetic Schemes for the Stereoselective Synthesis of Fraxinellone, Aryl Analogs Such as Analog 1, and Simplified Analog 2 Used in These Studies

A synthesis of fraxinellone was developed that would be short, stereoselective, and amenable to the production of analogs replacing the furan ring with other aryl groups, a key goal of our studies (Scheme 2). The synthesis takes advantage of the highly diastereoselective aldol reaction between enone **SM2** and aldehyde **SM1**, as reported by Fernández-Mateos.^31^ The racemic product was resolved using a kinetic resolution to provide intermediate **Int 1** in highly enantioenriched form (96:4 e.r.).^32^ Reduction of the alkene and conversion to the vinyl iodide **Int 2** set up an enabling Pd-catalyzed carbonylation to provide fraxinellone in enantioenriched form.^33^ Analog 1 could be made using an analogous sequence of steps starting from benzaldehyde **SM3**, providing the first aryl analog that serves as a control compound in our studies.^34^ Simplified analog 2 was discovered through testing of intermediates during our synthesis campaign and is readily available in 3 total steps, including a diastereoselective aldol of **SM5** lacking one methyl group and reduction of the alkene, as shown. These sequences provided access to the natural compound and many analogs straightforwardly, enabling extensive biological evaluation and identification of the enhanced activity of analog 2, as described below.

### Fraxinellone Analogs Protect against Glu Toxicity in PC12 and SH-SY5Y Cells

**Figure 2:**
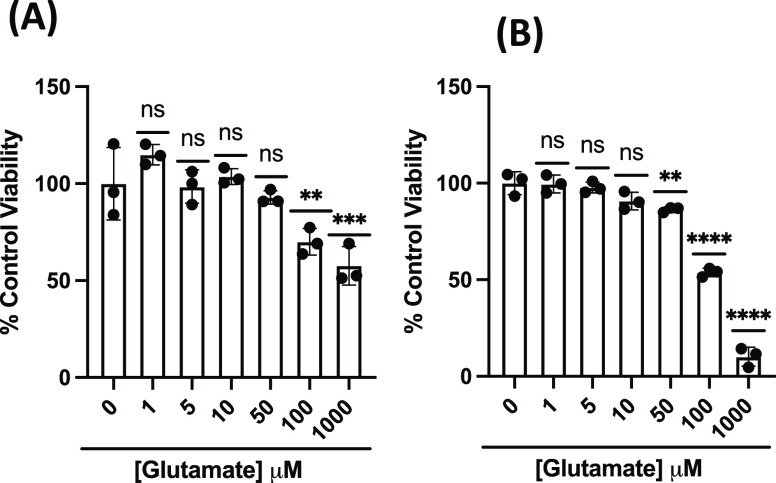
MTT analysis to assess cell viability in (A) PC12 and (B) SH-SY5Y cells treated with Glu for 24 h. Viability is shown as a percentage of PC12 or SH-SY5Y cells left untreated. Error bars show standard deviation (SD) for *n* = 3 replicates. **p < 0.05, ***p < 0.01 for ordinary one-way ANOVA comparing Glu-treated PC12 and SH-SY5Y cells to untreated cells with Dunnett correction for multiple comparisons.

Previous reports demonstrated that treatment with fraxinellone before Glu significantly protected against toxicity in vitro.^25^ We desired to explore these findings further and selected two different neuronal-like cell lines, i.e., PC12 (rat) and SH-SY5Y (human), as in vitro models. Initially, we used the 3-[4,5-dimethylthiazol-2-yl]-2,5-diphenyl tetrazolium bromide (MTT) assay to monitor the viability of both cell lines incubated with fraxinellone, analog 1, and analog 2 for 24 h to verify cell viability is minimally impacted at concentrations used in experiments. Twenty-four h treatment with 100 μM Glu reduced viability of PC12 cells by roughly 40–50% (Figure 2A) and SH-SY5Y cells by roughly 50% (Figure 2B), as measured by MTT.

**Figure 3:**
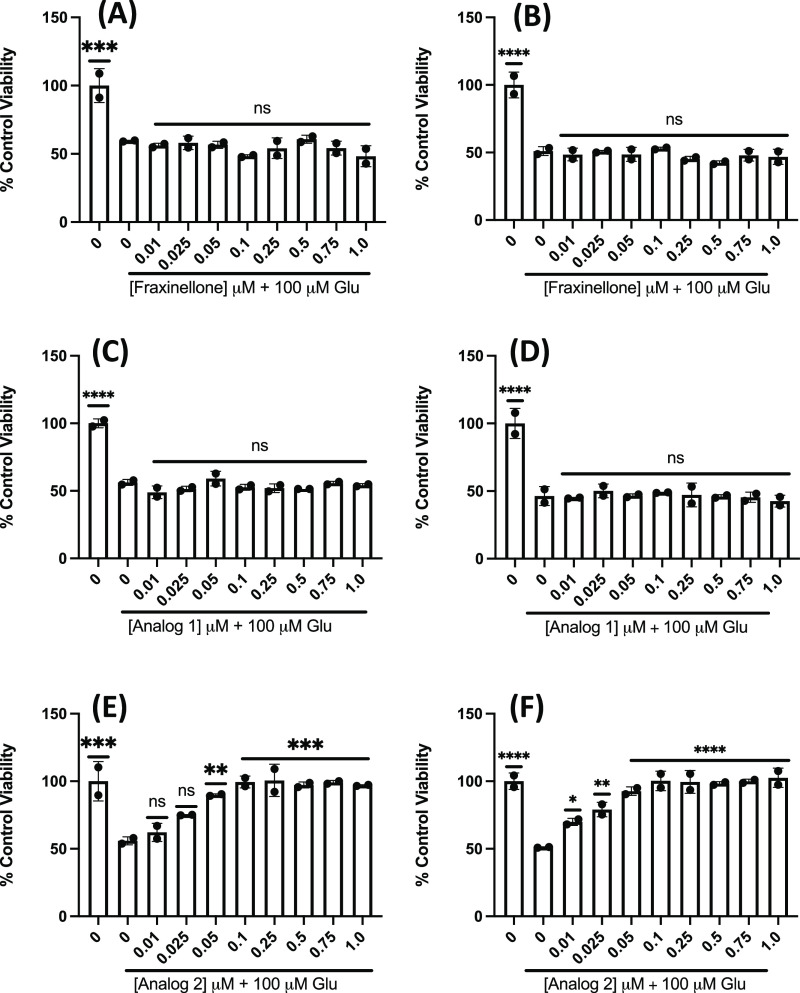
Pretreatment with analog 2 afforded protection against Glu-induced oxidative toxicity, whereas pretreatment with fraxinellone or analog 1 did not. MTT analysis to assess cell viability of (A) PC12 and (B) SH-SY5Y cells pretreated with a range of doses of fraxinellone for 30 min and then Glu (100 μM) for 24 h. MTT analysis to assess cell viability of (C) PC12 and (D) SH-SY5Y cells pretreated with a range of doses of analog 1 for 30 min and then Glu (100 μM) for 24 h. MTT analysis to assess cell viability of (E) PC12 and (F) SH-SY5Y cells pretreated with a range of concentrations of analog 2 for 30 min and then Glu (100 μM) for 24 h. Viability is shown as a percent of PC12 or SH-SY5Y cells left untreated. Error bars show standard deviation (SD) for *n* = 2 replicates. *p < 0.05, **p < 0.01, ***p < 0.0005, ****p < 0.0001 for ordinary one-way ANOVA comparing Glu-treated PC12 and SH-SY5Y cells to untreated cells with Dunnett correction for multiple comparisons.

Given this, we then sought to determine the activity of fraxinellone and several novel analogs against Glu-mediated toxicity (i.e., 100 μM Glu, 24 h) in PC12 and SH-SY5Y cells.^35^ A 30 min pretreatment with fraxinellone at ≤1 μM did not provide any significant protection against Glu toxicity in PC12 (Figure 3A) or SH-SY5Y cells (Figure 3B). Fraxinellone at significantly higher concentrations (i.e., μM range) mitigated Glu-mediated toxicity for PC12 cells (data not shown), as seen in previous reports.^26^ We also found that pretreatment with analog 1 did not protect against Glu toxicity in PC12 (Figure 3C) or SH-SY5Y (Figure 3D) cells; however, incubation with analog 2 before adding Glu yielded significant protection in a dose-dependent manner in both PC12 (Figure 3E) and SH-SY5Y cells (Figure 3F). For this analog, an EC_50_ of 44 and 39 nM was measured for PC12 and SH-SY5Y cells, respectively.

**Figure 4:**
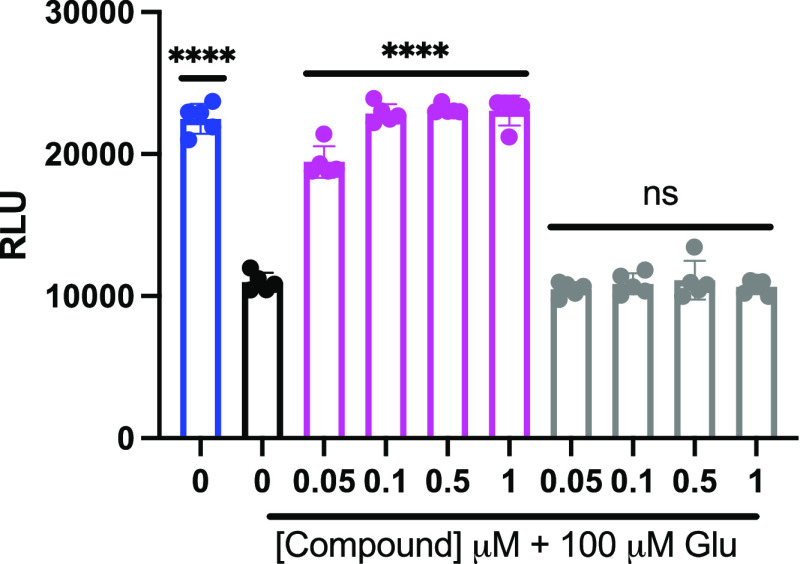
Pretreatment with analog 2 afforded protection against Glu-induced oxidative toxicity, whereas pretreatment with analog 1 did not. Cell-Titer Glo assay to assess viability of PC12 cells pretreated with a range of doses of analog 2 (Magenta) or analog 1 (Gray) for 30 min and then Glu (100 μM) for 24 h. Viability is shown as a percent of PC12 cells left untreated (Blue) and compared to cells incubated with Glu without either analog 1 or analog 2 (Black). Error bars show standard deviation (SD) for *n* = 5 replicates. ****p < 0.0001 for ordinary one-way ANOVA comparing Glu-treated PC12 cells to untreated cells with a Dunnett correction for multiple comparisons.

To enhance the rigor of the work, we repeated cell viability experiments using an assay for an alternative end point, i.e., ATP production. Using a commercial kit, cell viability (i.e., ATP) was measured following pretreatment with analog 2 (Magenta) or analog 1 (Gray) for 30 min, followed by Glu for 24 h in PC12 cells. This experiment yielded similar results to the MTT assay, as analog 2 (EC_50_ = 45 nM) provided significant protection against the Glu insult, but analog 1 did not (Figure 4). Of note, fraxinellone and its two analogs are washed from the cells following the 30 min incubation, thereby indicating significant cellular uptake and/or potent and rapid interaction with the molecular target(s).

### Fraxinellone Analog also Protects against the Toxicant Rotenone in PC12 Cells

**Figure 5:**
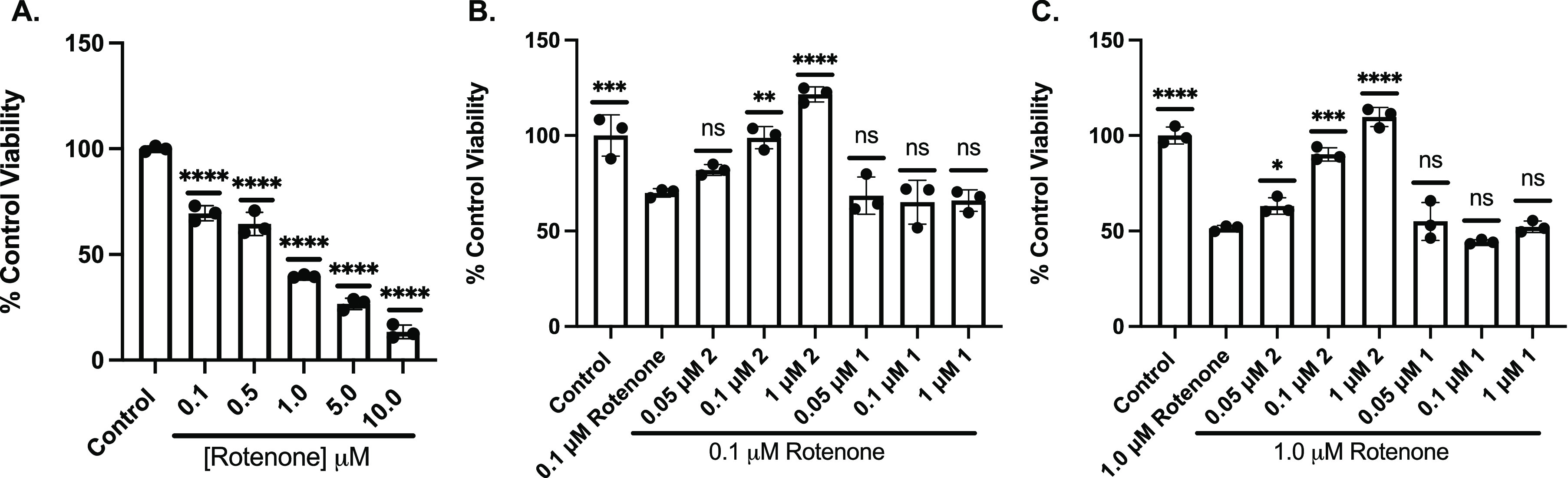
Pretreatment with analog 2 afforded protection against rotenone-induced oxidative toxicity, whereas pretreatment with analog 1 did not. MTT analysis to assess cell viability of PC12 treated with a range of doses of rotenone for 12 h (A). MTT analysis to assess cell viability of PC12 cells pretreated with analog 2 or analog 1 for 30 min and then 0.1 μM (B) or 1.0 μM (C) rotenone for 12 h. Viability is shown as a percent of PC12 cells left untreated. Error bars show standard deviation (SD) for *n* = 3 replicates. *p < 0.05, **p < 0.01, ***p < 0.0005, ****p < 0.0001 for ordinary one-way ANOVA comparing Rotenone ± analog 2 or 1 treated cells to untreated cells with Dunnett correction for multiple comparisons.

To test whether the protection afforded by the fraxinellone analog is generalizable and applicable to other oxidative insults, rotenone treatment was employed as a model toxicant using the same methods as previously reported.^28^ Rotenone is a neurotoxicant used in vitro and in vivo to model parkinsonism with a mechanism of action involving potent inhibition of Complex 1, yielding mitochondrial ROS and a loss of matrix NAD production.^36,37^ Cell viability was assessed following treatment with rotenone, a widely used pesticide, over a range of doses (Figure 5A). We then sought to investigate the activity of analog 2 and analog 1 against rotenone at a low (0.1 μM) (Figure 5B) and high (1 μM) (Figure 5C) dose. We pretreated our cells with analog 2 or analog 1 for 30 min and then washed cells before incubating with rotenone for 12 h.

Analog 2 significantly protected against 0.1 μM rotenone at as low as 0.05 μM, whereas analog 1 did not. Analog 2 also significantly protected against 1.0 μM rotenone at as low as 0.1 μM, whereas analog 1 did not. These results suggest a broader application of our fraxinellone analogs against different modulators of oxidative cell death, and in this case, one that targets the mitochondria (Complex 1); however, higher concentrations were needed to mitigate rotenone-mediated loss of cell viability compared to that seen following Glu treatment.

### Pretreatment with Analog 2 Enhances the Expression of Several Antioxidant Genes in a Time-Dependent Manner, a Key Mechanism of Nrf2 Activation

In reasoning a mechanism of action for the fraxinellone analog, we considered: (1) direct antioxidant activity; (2) Glu receptor antagonism; and (3) activation of the Nrf2 antioxidant response. Given the potency of the fraxinellone analog (i.e., 44 and 39 nM) in the presence of an excess of Glu (i.e., 100 μM), we thought it was unlikely that direct antioxidant activity played a key role in its mechanism of protection. In addition, the analog’s structure does not feature common antioxidant motifs, such as a free thiol, hydroxylated chromane ring, or polyphenol.^38^ With the lack of a nitrogen atom, it seemed unlikely that the fraxinellone analog would be an effective Glu receptor antagonist. The structure of the analog appeared dissimilar to other known Glu antagonists, such as ketamine, memantine, and riluzole.^39^

Based on this rationale, we decided to investigate the possibility that Nrf2 activation was a mechanistic target for the fraxinellone analog. The antioxidant response is primarily regulated by the transcription factor Nrf2 binding to the antioxidant response element (ARE) sequences within the promoter region of target genes to induce their expression.^16^ Knowing analog 2 potently and rapidly mitigates Glu-mediated ROS production, we sought to determine whether the mechanism of action of analog 2 involved activation of Nrf2, whether upstream or downstream. qPCR was used to show that analog 2 induces expression of Nrf2 target genes, including *Gpx4*, *Nqo1*, and *Sod1*, whereas inactive analog 1 did not, as a control.^40^

**Figure 6:**
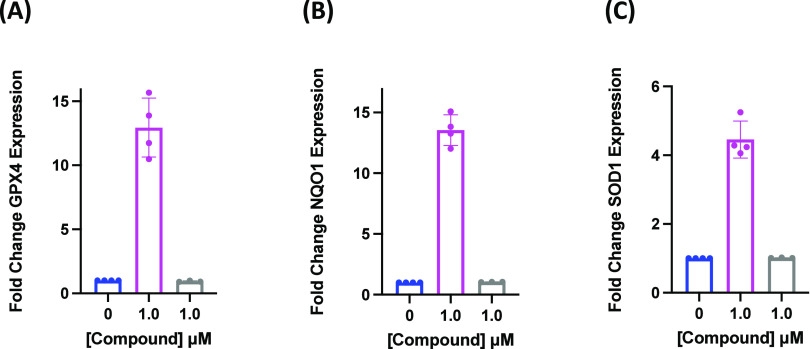
Pretreatment with analog 2 alone induces upregulation of Nrf2-dependent oxidative stress response genes in PC12 cells after 4 h. Expression of Nrf2 target genes, (A) *Gpx4*, (B) *Nqo1*, and (C) *Sod1*, as measured by qPCR, in PC12 cells pretreated with analog 2 (Magenta) or analog 1 (Gray) (0.05 or 1.0 μM) for 4 h. Error bars show for SEM for *n* = 3 replicates.

**Figure 7:**
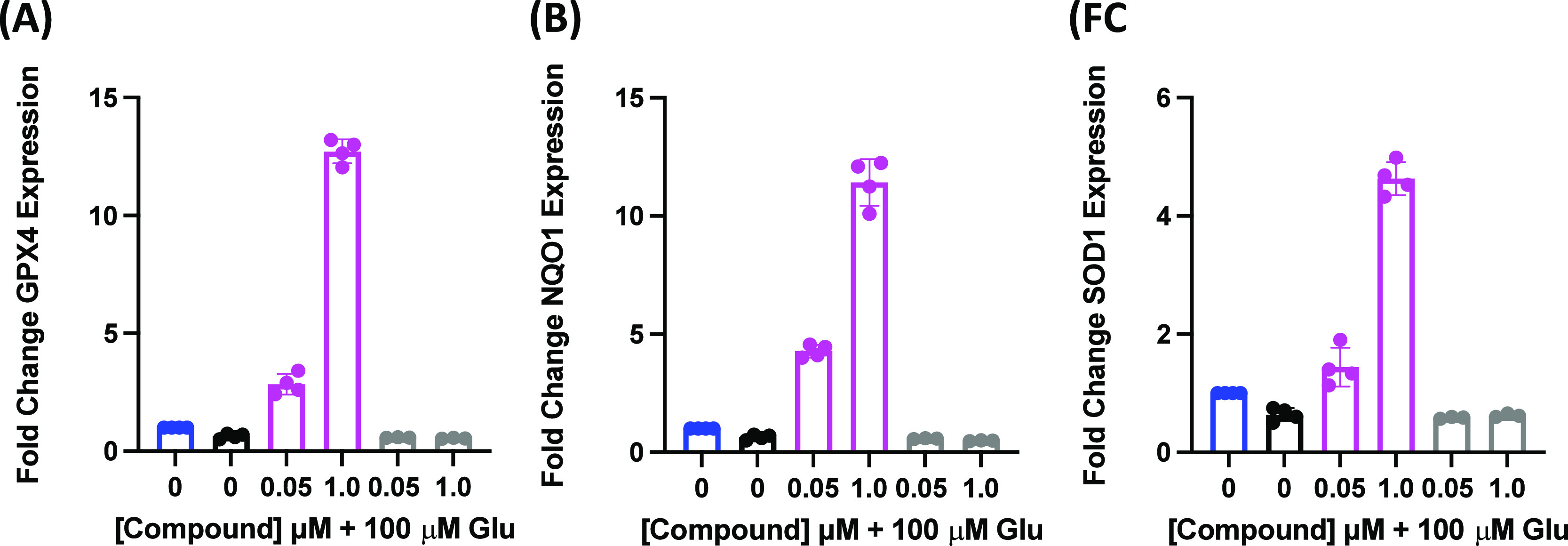
Pretreatment with analog 2 followed by 4 h Glu exposure induces upregulation of Nrf2-dependent oxidative stress response genes in PC12 cells. Expression of Nrf2 target genes, (A) *Gpx4*, (B) *Nqo1*, and (C) *Sod1*, as measured by qPCR, in PC12 cells pretreated with analog 2 (Magenta) or analog 1 (Gray) (0.05 or 1.0 μM) before Glu (100 μM) for 4 h. Error bars show for SEM for *n* = 4 replicates.

**Figure 8:**
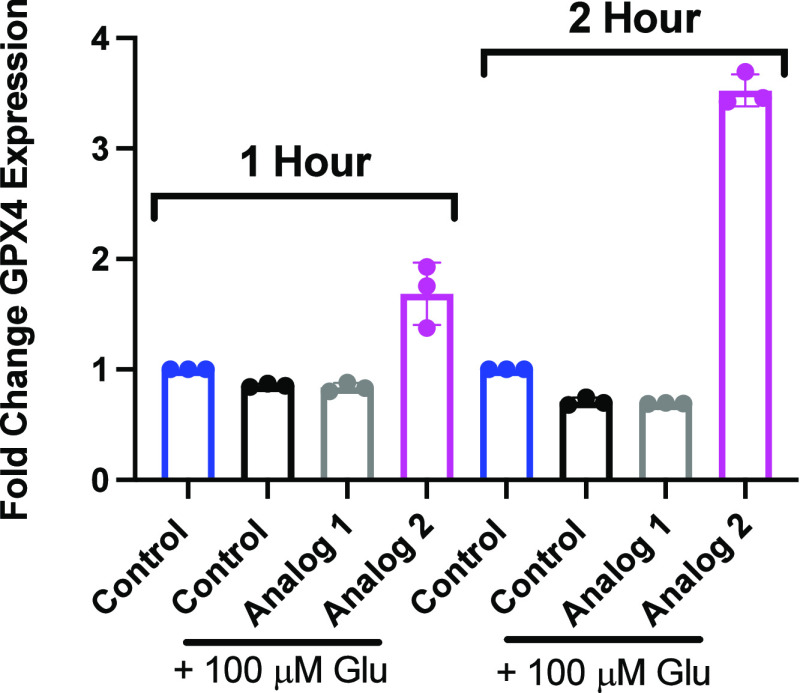
Upregulation of Nrf2-related genes is time-dependent. Induction of GPX4 expression is seen in PC12 cells pretreated with 100 nM analog 1 or 2 followed by 1 or 2 h exposure to 100 μM Glu. Expression of Nrf2 target gene *Gpx4*, as measured by qPCR, in PC12 cells pretreated with analog 1 (Gray) or analog 2 (Magenta) before Glu for 1 or 2 h. Error bars show SEM for *n* = 3 replicates.

Incubation with analog 2 (Magenta) alone significantly increased expression of *Gpx4* (Figure 6A), *Nqo1* (Figure 6B), and *Sod1* (Figure 6C) after 4 h, whereas analog 1 (Gray) did not. Pretreatment with analog 2 (Magenta), followed by incubation with Glu for 4 h increased expression of *Gpx4* (Figure 7A), *Nqo1* (Figure 7B), and *Sod1* (Figure 7C) whereas analog 1 (Gray) did not. Lastly, pretreatment with analog 2 (Magenta), followed by Glu for 1 or 2 h enhanced expression of *Gpx4* (Figure 8) in a time-dependent manner, whereas analog 1 (Gray) did not. As noted in Figure 8, the increase in gene expression was seen as early as 1 h following incubation. These data indicate analog 2 strongly and rapidly enhances ARE expression alone or in response to elevated levels of Glu, with the mechanism of action likely through Nrf2 activation, and in a time-dependent manner. To confirm the induction of protein expression, Western blot analysis was performed demonstrating treatment of PC12 cells with analog 2 but not analog 1 yielded increased levels of GPX4 (Supporting Information Figure S1).

### Fraxinellone Analog Mitigates Time-Dependent ROS Production Following Glu Treatment

**Figure 9:**
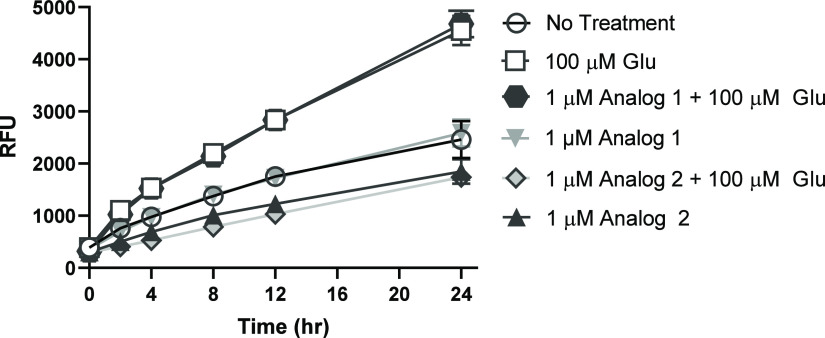
Glu-mediated ROS production is attenuated by analog 2. Data shown are for mean H2DCFDA fluorescence of PC12 cells treated with Glu, pretreated with analog 1 followed by Glu, pretreated with analog 2 followed by Glu, pretreated with analog 1, pretreated with analog 2 only, or left untreated. Error bars show SEM for *n* = 12 replicates

We next sought to determine if analog 2 reduces Glu-mediated ROS production in PC12 cells, thereby, providing cytoprotection.^41^ In untreated cells, accumulation of ROS occurred throughout 24 h as measured using 2′,7′-dichlorodihydrofluoresceine diacetate (DCFDA) (Figure 9). PC12 cells showed a significant increase in ROS as early as 4 h following Glu exposure alone. Treatment with analog 1 before Glu did not attenuate oxidative stress in Glu-treated cells; however, incubation with analog 2 before the Glu insult significantly attenuated ROS production as early as 2 h, indicating analog 2 rapidly reduces oxidative stress caused by Glu. Interestingly, treatment with analog 2 alone (in the absence of excess Glu) also significantly reduced basal ROS production in PC12 cells as early as 2 h, whereas analog 1 did not.

**Figure 10:**
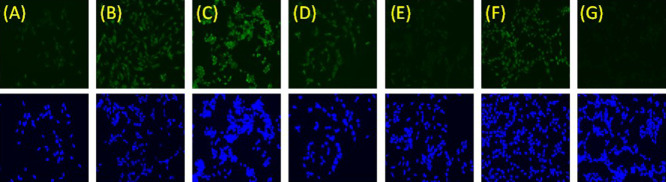
Preincubation (30 min) with SFN or analog 2 mitigates production of ROS in SH-SY5Y cells treated with 100 μM Glu for 4 h. Show are images of cells costained with both CellROX (top) and NucBlue (bottom) following 4 h of incubation: (A) untreated control; (B) 100 μM Glu; (C) 10 μM menadione (positive control); (D) 100 μM Glu + 1 μM SFN; (E) 100 μM Glu + 10 μM SFN; (F) 100 μM Glu + 100 nM Analog 1; and (G) 100 μM Glu + 100 nM Analog 2.

To visualize ROS production and mitigation via fraxinellone and analogs, we pretreated SH-SY5Y cells with 1 or 10 μM SFN or 100 nM analog 1 or analog 2 for 30 min before incubation with 100 μM Glu. ROS was detected via CellROX after 4 h with 100 μM Glu or 10 μM menadione (positive control). As shown in Figure 10, pretreatment with either 10 μM SFN (Figure 10E) or 100 nM analog 2 (Figure 10G) yielded a significant reduction in CellROX staining, even less than that observed for the negative control (cells only).

### Cytoprotective Activity of the Fraxinellone Analog Can Be Replicated Using SFN, a Nrf2 Activator, and Antagonized Using ML-385, a Nrf2 Inhibitor

**Figure 11:**
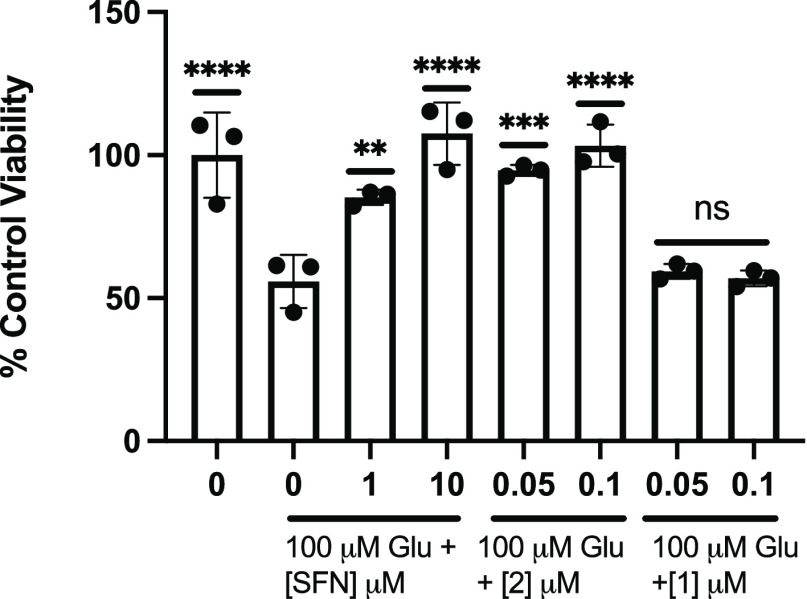
SFN protects against Glu excitotoxicity but at higher concentrations when compared to analog 2. MTT analysis of treatment with analog 2 or 1 (50 or 100 nM) or SFN (1 and 10 μM) followed by Glu for 24 h. Viability is shown as a percent of PC12 cells left untreated. Error bars show standard deviation (SD) for *n* = 3 replicates. **p < 0.01, ***p < 0.0005, ****p < 0.0001 for ordinary one-way ANOVA comparing Glu-treated PC12 cells to untreated, SFN, analog 2 or 1 treated cells with Dunnett correction for multiple comparisons.

Nrf2 activity is regulated through its interaction with the redox sensor E3 ligase protein KEAP1. KEAP1 promotes the ubiquitination of Nrf2 in the absence of oxidative stress; however, in response to inflammatory, environmental, or oxidative stressors, sensor Cys residues on KEAP1 are covalently modified by these stressors, thus stabilizing Nrf2 to promote its transcriptional activity.^16,42^ A notable Nrf2 activator is SFN, an isothiocyanate that readily induces KEAP1-Nrf2 signaling and enhances ARE gene expression.^43^ We used SFN as a positive control for protection against Glu excitotoxicity through Nrf2 activation and desired to compare its potency to the fraxinellone analogs. Results in Figure 11 represent outcomes of experiments involving pretreatment with SFN or fraxinellone analogs before the addition of 100 μM Glu for 24 h. As shown in Figure 11, SFN replicated the protective activity of the fraxinellone analog; however, higher concentrations of SFN were required (1 and 10 μM) for significant protection, compared to the analog (50 and 100 nM). Analog 1 showed no activity. In addition, to achieve measurable and significant activity of SFN, the treatment protocol needed to be modified to remove the wash step before the 30 min pretreatment. Washing the cells before the addition of 100 μM Glu significantly attenuated the protective activity of SFN.

### Nrf2 Inhibitor ML-385 Antagonizes the Activity of Analog 2

**Figure 12:**
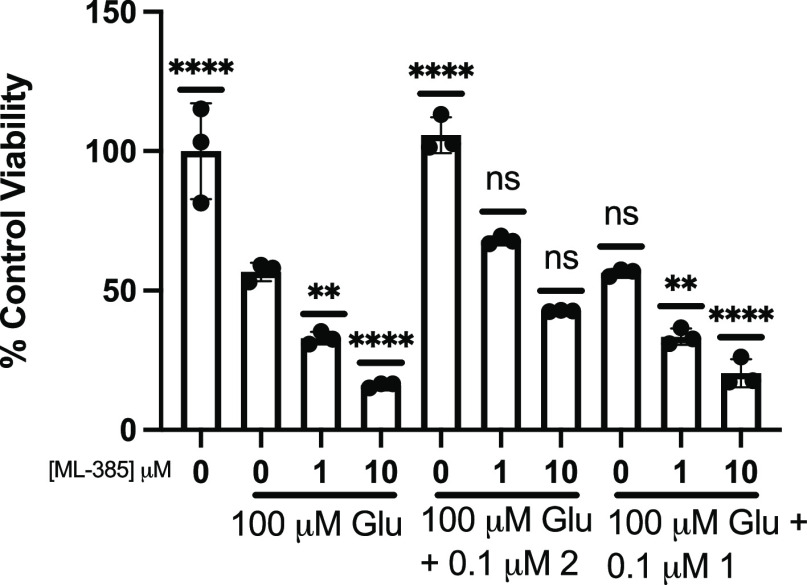
ML-385 antagonized the activity of analog 2 in the presence of Glu. MTT analysis of treatment with analog 2 (50 or 100 nM) ± ML-385 (1 or 10 μM), analog 1 (50 or 100 nM) ± ML-385 (1 or 10 μM), or ML-385 (1 and 10 μM) followed by Glu for 24 h. Viability is shown as a percent of PC12 cells left untreated. Error bars show standard deviation (SD) for *n* = 3 replicates. **p < 0.01, ***p < 0.0005, ****p < 0.0001 for ordinary one-way ANOVA comparing Glu-treated PC12 cells to untreated and ML-385 ± analog 2 or 1 treated cells with Dunnett correction for multiple comparisons.

Upon activation, Nrf2 readily accumulates in the cytosol, followed by translocation to the nucleus. Once in the nucleus, Nrf2 interacts with sMaf proteins to form the Nrf2-MafG protein complex, which is required for Nrf2 to bind ARE and induce the expression of antioxidant genes. ML-385 is a Nrf2 inhibitor that blocks downstream ARE-mediated gene expression by disrupting the formation of the Nrf2-MafG complex, thereby preventing the binding of Nrf2 to the ARE.^44^ To further demonstrate the involvement of Nrf2 activation in the mechanism of action of the fraxinellone analog, we employed ML-385 in experiments to determine whether or not it antagonized the protective activity of the analog. As shown in Figure 12, pretreatment with ML-385 significantly attenuated protection toward Glu excitotoxicity afforded by the fraxinellone analog. Interestingly, ML-385 augmented the toxicity of Glu, likely by antagonizing the basal Nrf2 response.

### Treatment with Analog 2 Induces Nuclear Translocation of Nrf2 and Binding to the ARE, Likely in a Thiol-Independent Manner

**Figure 13:**
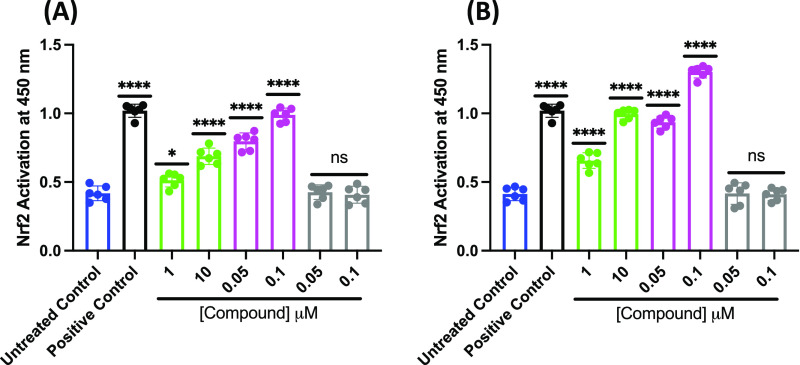
Analog 2 and SFN induce Nrf2 nuclear translocation in PC12 cells, whereas analog 1 does not. PC12 cells were treated with SFN (1 or 10 μM; Green), analog 2 (50 or 100 nM; Magenta) or analog 1 (50 or 100 nM; Gray) or left untreated (Blue) for (A) 1 h or (B) 4 h. A positive control was included for comparison (Black). Nuclear lysates were prepared and levels of active Nrf2 binding to DNA were measured. Error bars show standard deviation (SD) for *n* = 3 replicates. **p < 0.01, ***p < 0.0005, ****p < 0.0001 for ordinary one-way ANOVA comparing SFN, analog 2, or analog 1 to untreated treated cells with Dunnett correction for multiple comparisons.

To further elucidate the mechanism of action for analog 2, we assessed the Nrf2 DNA-binding activity from cells treated with SFN, analog 2, and analog 1. Following incubation with SFN, analog 2, or analog 1 for 1 h (Figure 13A) or 4 h (Figure 13B) hr, enhancement of Nrf2 nuclear translocation and DNA-binding is seen in cells treated with the positive control, SFN (1 and 10 μM), and analog 2 (50 and 100 nM). Nrf2 translocation is greater in cells treated with 100 nM analog 2 for 4 h when compared to the positive control (SFN). Enhancement of Nrf2 translocation is also seen in cells treated with 1 and 10 μM SFN at both 1 and 4 h, but not as great as compared to analog 2. Treatment with analog 1 at both time points yielded similar results to the untreated control, indicating no activation of Nrf2. These findings demonstrate that treatment of cells with the fraxinellone analog yields Nrf2 nuclear translocation and binding to the ARE.

### Analog 2 Likely Activates Nrf2 in a Thiol-Independent Manner

**Figure 14:**
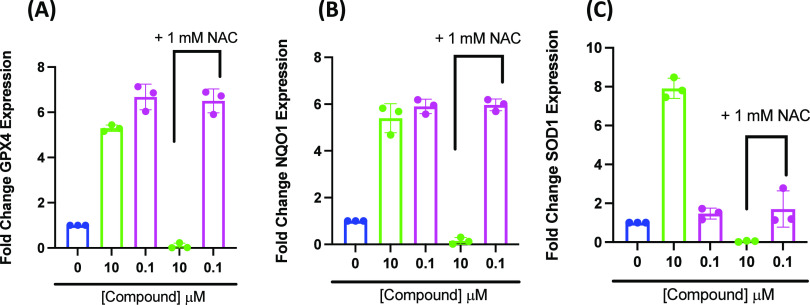
Treatment with SFN or analog 2 induces upregulation of Nrf2-dependent oxidative stress response genes in PC12 cells after 4 h. When cells are pretreated with NAC, SFN induced upregulation of Nrf2-dependent AREs is eliminated; however, induction is seen for cells treated with analog 2. Expression of Nrf2 target genes was measured: (A) *Gpx4*, (B) *Nqo1*, and (C) *Sod1* in PC12 cells pretreated with 10 μM SFN (Green) or 100 nM analog 2 (Magenta) ± 1 mM NAC for 4 h and compared to a negative control not incubated with either SFN or analog 2 (Blue). Error bars show the SEM for *n* = 3.

N-acetylcysteine (NAC) is a reactive thiol scavenger that serves as a precursor to l-cysteine, resulting in elevated biosynthesis of glutathione.^45,46^ To determine the thiol-dependence for the protective activity of the fraxinellone analog, we treated our cells with 1 mM NAC for 1 h, followed by incubation with SFN or the analog for 4 h. Treatment with SFN or analog 2 alone increased expression of *Gpx4* (Figure 14A), *Nqo1* (Figure 14B), and *Sod1* (Figure 14C) after 4 h; however, pretreatment with 1 mM NAC for 1 h completely antagonized gene expression by SFN but did not affect the activity of the fraxinellone analog. Since NAC readily reacts with thiols, it was predicted NAC would readily react with SFN, although it was unknown whether or not the thiol would block gene expression by the fraxinellone analog. Such data suggest the fraxinellone analog is acting via a thiol-independent mechanism.

We established that a novel fraxinellone analog protects against both endogenous (i.e., Glu excitotoxicity) and exogenous (i.e., rotenone) toxicants through Nrf2 activation at measured EC_50_ values in the nM range. These data indicate Analog 2 has the potential to protect against multiple neurotoxicants; however, higher concentrations were needed to mitigate toxicity from rotenone compared to Glu. The difference in apparent potency of Analog 2 for Glu and rotenone likely stems from the mechanisms of action for each insult showing similarities, such as oxidative stress, but also several differences. While the high level of Glu produces intracellular ROS formation, the model parkinsonian neurotoxicant, rotenone, inhibits Complex 1, thereby impairing respiration and generating ROS, both detrimental to the cell. As noted in Figure 9, Analog 2 potently inhibits the production of ROS via Glu, and therefore, toxicants that act via ROS generation may be targets of this analog. Future work should determine the range of protective activity afforded by Analog 2.

Following the identification of an active analog (Analog 2) mitigating Glu-mediated toxicity, we sought to determine the mechanism of action, considering Analog 2 does not structurally resemble an antioxidant or Glu antagonist.^38,39^ About the latter point (i.e., Glu antagonist), we found that Analog 2 is also effective against the parkinsonian neurotoxicant rotenone and not just Glu. In addition, a short incubation (30 min) with the analog is needed to afford protection, suggesting rapid activation of defense factors, whereas direct antioxidant activity would likely take longer. Given these findings, we postulated Nrf2-dependent signaling as a mechanism, although Analog 2 does not have the usual features of an Nrf2 activator (i.e., thiol modifier or oxidizer). We found that the active compound rapidly induces the expression of Nrf2-dependent genes *Gpx4*, *Nqo1*, and *Sod1*, thus decreasing Glu-mediated ROS levels in a time-dependent manner. These results were corroborated as we could replicate protection via a known Nrf2 activator, SFN, and block Analog 2-mediated protection via ML-385, an inhibitor of the Nrf2-MafG complex binding the ARE (Figure 11). Interestingly, SFN only protected against the Glu insult when not washed from our cells and at much higher concentrations (i.e., 10 μM) when compared to our analog (i.e., nM range). The findings were further confirmed via the use of a Nrf2 translocation assay (Figure 13), and of note, Analog 2 appeared to be more effective inducing Nrf2 translocation compared to SFN.

At this point, the mechanism of action via which Analog 2 activates the Nrf2 cellular defense is not known. It is conceivable that the active analog is metabolized or rearranges to a thiol-reactive intermediate (e.g., epoxide or α,β-unsaturated carbonyl), for example, furans are known to be metabolized via cytochrome P450 to a reactive epoxide (Figure S2);^47^ however, the low concentrations (nM range) and short treatment time frame (30 min) utilized cast doubt on such possibility. Furthermore, we found that pretreatment of the cells with 1 mM NAC completely blocked the induction of Nrf2 target genes by SFN but had no effect on Analog 2 (Figure 14), suggesting thiol-dependence for SFN but not Analog 2. Additionally, it is conceivable that the active analog noncovalently inhibits the Nrf2-Keap1 protein–protein interaction, which has been demonstrated for several agents, including natural products.^48^ A noncovalent activator of the Nrf2 cellular defense would present opportunities for therapeutic development and minimize potential liabilities (e.g., poor pharmacokinetics and off-target protein binding) for covalent-modifiers of Keap1. Future work will further probe the mechanism of action for Analog 2.

## Methods

### Analog Synthesis

Fraxinellone, analog 1, and analog 2 were synthesized via a new synthetic route starting from 2,6-dimethylcyclohexanone or cyclohexenone. Key steps involved a diastereoselective aldol reaction with 3-furaldehyde, alkene reduction, formation of a vinyl iodide, and lactone formation. Details of the synthesis and characterization data for all new intermediates and analogs can be found in the Supporting Information.

### Cell Culture

PC12 rat cells were obtained from American Type Culture Collection (Manassas, VA, U.S.A.) and were grown in tissue culture flasks in RPMI 1140 supplemented with 10% horse serum, 5% fetal bovine serum (FBS), and 1% penicillin/streptomycin at 37 °C in a humidified atmosphere of 5% CO_2_. All experiments on these cells were performed between passages 9–14 to reduce interexperimental variability. All cells were treated with equal volumes of DMSO prior to harvesting for analysis.

SH-SY5Y human neuroblastoma cells were obtained from American Type Culture Collection (Manassas, VA, U.S.A.) and were grown in Opti-MEM supplemented with 10% FBS, 1% MEM-nonessential amino acids, 1% penicillin/streptomycin, and 1 mM sodium pyruvate at 37 °C in a humidified atmosphere of 5% CO_2_. All experiments on these cells were performed between passages 8–10 to reduce interexperimental variability. All cells were treated with equal volumes of DMSO prior to harvesting for analysis.

### Cell Viability Assays

Cell viability was assessed using Cell-Titer Glo reagent (Promega) or the colorimetric reagent 3-[4,5-dimethylthiazol-2-yl]-2,5-diphenyl tetrazolium bromide (MTT) (Sigma-Aldrich). Cell-Titer Glo was performed following the manufacturer’s protocol. For MTT analysis, cultures were incubated in HBSS/glucose with 2 mg/mL MTT for 2 h at 37 °C. Following incubation, 0.4 mL of DMSO was added to each well to solubilize the formazan product. Reduced MTT was measured on a microplate reader (Molecular Devices Spectra Max 190) at 570 nm with a reference of 650 nm.

### In Vitro Glu Excitotoxicity and Rotenone Assays

Cells were pretreated with fraxinellone, analog 1, or analog 2 at a range of 0–1 μM for 30 min. The analogs were then washed from the cells before adding Glu (100 μM final concentration) for 24 h.^25^ Cell-Titer Glo and MTT were both used to assess the cell viability.

Cells were pretreated with analog 1 or analog 2 at a range of 0–1 μM for 30 min. The analogs were then washed from the cells before adding a low (0.1 μM) or high (1.0 μM) final concentration of rotenone for 12 h. MTT analysis was performed to assess cell viability.

### Quantification of ROS

Cells were collected, resuspended in 10 mL of fresh media containing 25 μM DCFDA, and incubated for 30 min in this solution. DCFDA was then washed off of the cells. The cells were then plated in a 96-well black plate, nontreated (Thermo Fisher Scientific) and treated, as described above. Images were taken on a BioTek Synergy 2 using ex. 485 nm and em. 535/30 channel at 0, 2, 4, 8, 12, and 24 h.

### Qualitative Assessment of ROS via Live Cell Imaging

ROS production was detected using a CellROX green reagent (Thermo Fisher). Briefly, SH-SY5Y cells were stained with 5 μM CellROX for 30 min prior to 30 min treatment with 1 or 10 μM SFN, or 100 nM Analog 1 or Analog 2, and then exposure to 100 μM Glu for 4 h. In addition, cells were incubated with 10 μM menadione as a positive control for ROS production. Following treatment, live cell images were taken to measure ROS production using an Evos FL Auto 2 (Thermo Fisher). Cells were stained 15 min prior to imaging with the NucBlue Live ReadyProbes Reagent.

### Quantitative PCR (qPCR)

PC12 cells were subjected to two different experimental conditions. Cells were (1) pretreated with analog 2, analog 1, or DMSO vehicle for 30 min before Glu for 1, 2, or 4 h; (2) treated with SFN or analog 2 ± NAC. Cells were then lysed, and total RNA was extracted using the RNeasy Mini Kit (Qiagen) according to the manufacturer’s instructions. RNA yield was quantified using Nanodrop (Thermo Fisher). cDNA was generated from 2 μg using a High-Capacity cDNA Reverse Transcription Kit (Applied Biosystems, cat. 4311235). qPCR reactions were prepared using PowerUp SYBR Green PCR Master Mix (Applied Biosystems) and primers (Table S1) obtained from Integrated DNA Technologies. Amplifications were run in an Applied Biosystems QuantStudio 3 plate reader with an initial melting period of 95 °C for 2 min and then 40 cycles of 2 min at 50 °C, 2 min at 95 °C, 15 s at 95 °C, 15 s at 58 °C, 1 min at 72 °C, 15 s at 95 °C, 1 min at 60 °C, and 15 s at 95 °C. Housekeeping genes used to normalize the control were TBP and β-actin (Supporting Information Table S1).

### Nrf2 Activation and Inhibition

SFN (Cayman Chemicals), an Nrf2 activator, was used for the present experiments. Cells were treated with either 1 or 10 μM SFN followed by Glu for 24 h, or 50 or 100 nM analog 1 or analog 2 followed by Glu for 24 h before MTT analysis was used to assess cell viability.

ML-385 (Cayman Chemicals), a Nrf2 inhibitor, was used for the present experiments. Cells were treated with either 1 or 10 μM ML-385 followed by Glu for 24 h, 50 nM or 100 nM analog 2 or analog 1 followed by Glu for 24 h, 50 nM or 100 nM analog 2 or analog 1 followed by 1 or 10 μM ML-385 and then Glu for 24 h before MTT analysis was performed to assess cell viability.

### Nrf2 DNA-Binding Assay (Transcription Factor Assay)

Cells were treated with 1 or 10 μM SFN (positive control), 50 or 100 nM analog 1 or analog 2, or left untreated (negative control) for 1 or 4 h. Following treatment, nuclear protein extracts were prepared using the Nuclear Extract Kit (Active Motif), according to the manufacturer’s protocol. Nrf2 DNA-binding activity of nuclear extracts was performed using the TransAM Nrf2 ELISA-based transcription factor assay kit (Active Motif) containing a 96-well plate with immobilized ARE oligonucleotides, according to the manufacturer’s protocol.

### Western Blot Analysis

Cells were treated with 1 or 10 μM SFN, 100 nM Analog 1 or Analog 2, or no treatment for 4 h before the cell lysate was collected. Briefly, cells were prepared in a complete lysis buffer composed of 0.1% 1 M DTT, 1.0% protease inhibitor cocktail, and 99% lysis buffer (Active Motif) along with brief sonication. Protein concentration was quantified using the Bradford Assay (Pierce). The positive (+) control represents the cell lysate from mouse tissue (cardiomyocytes) known to express GPX4. A total of 5 μg of protein was loaded for electrophoretic separation on a SDS-polyacrylamide gel and then transferred onto a PVDF membrane. Following incubation with 5% bovine serum albumin (BSA) at room temperature for 1 h, membranes were then incubated with primary antibody anti-GPX4 (ab125066, Abcam) at 4 °C overnight and then washed. Horseradish peroxidase-conjugated secondary antibody was then added, incubated in the dark at room temperature for 2 h, and washed. Images were taken with an iBright FL1000 (Thermo Fisher).

## Supporting Information Available

Western blot for GPX4, metabolic bioactivation of Analog 2, forward and reverse primers, general methods for synthetic chemistry and synthesis of fraxinellone analogs, and NMR spectra (PDF)

## Abbreviations

ARE: antioxidant response elements
DCFDA: 2′,7′-dichlorodihydrofluoresceine diacetate
GPX4: glutathione peroxidase
Maf: musculoaponeurotic fibrosarcoma
NAC: *N*-acetylcysteine
NQO1: NAD(P)H quinone oxidoreductase
Nrf2: NF-E2-related factor 2
ROS: reactive oxygen species
SFN: sulforaphane
SOD1: superoxide dismutase
