# Supplementary material for: A Novel Analog of the Natural Product Fraxinellone Protects against Endogenous and Exogenous Neurotoxicants
Source: ACS Chem Neurosci. 2024 Jun 26;15(14):2612–22. doi: 10.1021/acschemneuro.4c00090 (PMC11258694; doi:10.1021/acschemneuro.4c00090)
Supplement: Supplementary file 1 — cn4c00090_si_001.pdf [file cn4c00090_si_001.pdf]

Supporting Information

A Novel Analog of the Natural Product Fraxinellone Protects Against Endogenous and Exogenous Neurotoxicants

Anna E. Bartman[1], Mersad Raeisi[2], Clarence D. Peiris[2], Isabella E. Jacobsen[2], David B.C. Martin[2,*], Jonathan A. Doorn[1,*]

Email: ***jonathan-doorn@uiowa.edu***, ***david-martin@uiowa.edu***

[1]Department of Pharmaceutical Sciences & Experimental Therapeutics, College of Pharmacy, University of Iowa, Iowa City, Iowa 52242, United States.

[2]Department of Chemistry, College of Liberal Arts & Sciences, University of Iowa, Iowa City, Iowa 52242, United States.

Table of Contents

Supplementary Figures **S3**

General Methods for Synthetic Chemistry: **S5**

Synthesis of fraxinellone and analogs **S6**

- **Scheme S1: Enantioselective synthesis of (–)-fraxinellone** **S6**
- **Scheme S2: Synthesis of Analog 1** **S10**
- **Scheme S3: Synthesis of Analog 2** **S12**

NMR Spectra **S15**

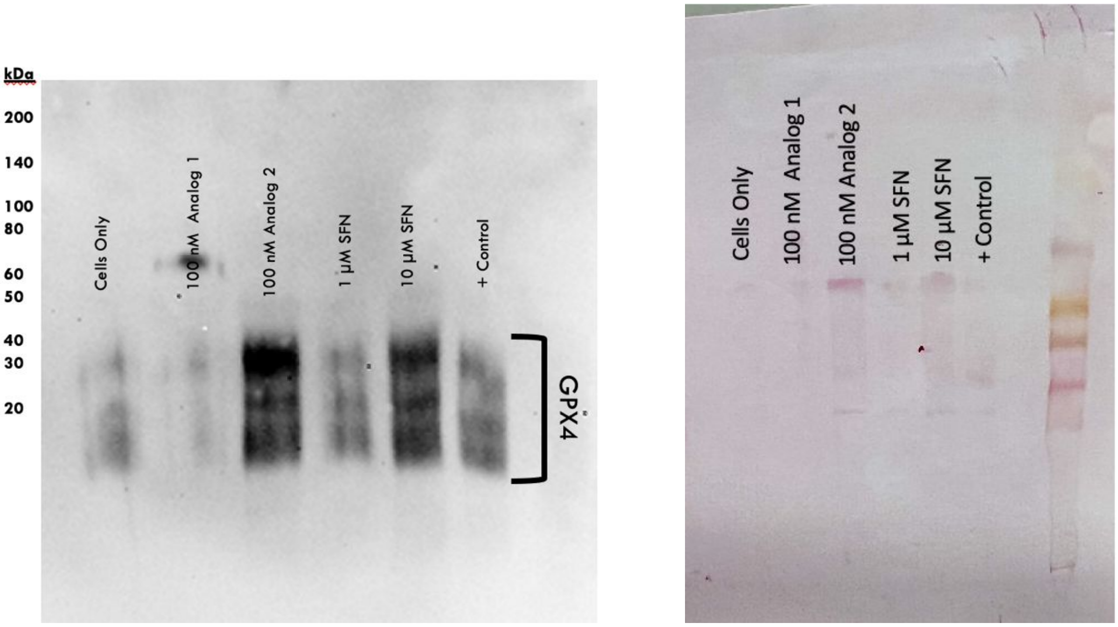

Supplementary Figure S1. Western Blot and Ponceau Red staining to confirm expression of GPX4 protein in PC12 cells. Cells were treated with 1 or 10 μM SFN, 100 nM Analog 1 or Analog 2, or no treatment for 4 hr.

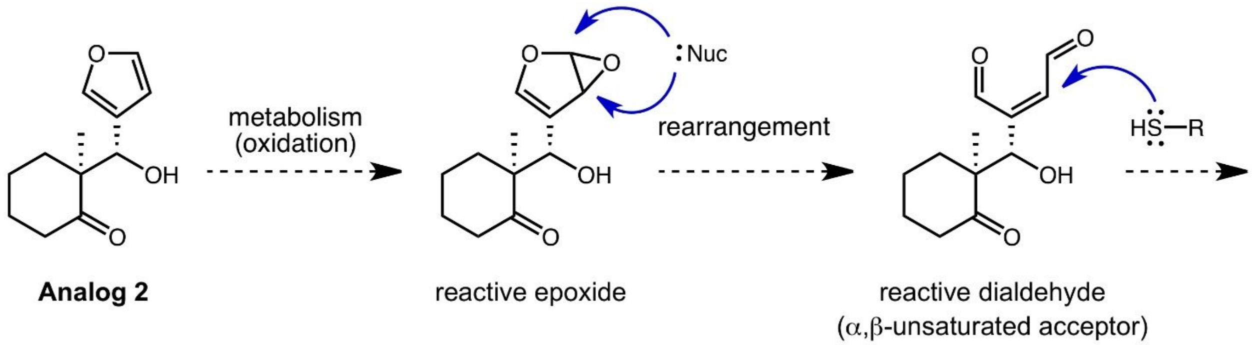

Reference: Peterson, L.A. Reactive Metabolites in the Biotransformations of Molecules Containing a Furan Ring. *Chem. Res. Toxicol.* **2013**, *26*, 6-25.

Supplementary Figure S2. Metabolic bioactivation of Analog 2 could produce an epoxide which rearranges to a thiol-reactive α,β-unsaturated carbonyl.

Table S1: Forward and Reverse Primers

Gene	Forward Primer	Reverse Primer
GPX4	5’-GCAACCAGTTTGGGAGGCAGGAG-3’	5’-CCTCCATGGGACCATAGCGCTTC-3’
NQO1	5’-GCGTCTGGAGACTGTCTGGG-3’	5’-CGGCTGGAATGGACTTGC-3’
SOD1	5’-GCAGAAGGCAAGCGGTGAAC-3’	5’-TAGCAGGACAGCAGATGAGT-3’
β-actin	5’-AAGATCCTGACCGAGCGTGG-3’	5’-CAGCACTGTGTTGGCATAGAGG-3’
TBP	5’-CGTGACGATAACCCAGAAAG-3’	5’-GGTGGAAGGCTGTTGTTC-3’

General Methods for Synthetic Chemistry:

All reactions were carried using oven dried or flame dried glassware charged with a magnetic stir bar and conducted under an inert nitrogen atmosphere using typical Schlenk techniques, unless otherwise noted. Solvents were dried by passage through columns of activated alumina or distilled and stored under nitrogen over 5 Å sieves or otherwise freshly distilled. All starting materials were prepared according to known literature procedures or used as obtained from commercial sources, unless otherwise indicated. Aldehydes were freshly distilled prior to use. Reactions were monitored by thin-layer chromatography (TLC) and carried out on 0.25 mm coated commercial silica gel plates (Analtech TLC Uniplates, F254 precoated glass plates) using UV light as visualizing agent and/or $KMnO_4$ and heat as a developing agent. Unless otherwise indicated, silica gel chromatography was performed using flash chromatography on P60 silica. Alternatively, a Yamazen Smart Flash AI-580S system in conjunction with Yamazen Universal Premium 40g columns with the specified gradient elution mode was used when indicated.

^{1}H and ^{13}C NMR spectra were recorded on a Bruker Avance NEO 400, Bruker Avance NEO 500, or Bruker Avance III 600 MHz spectrometer and were internally referenced to residual protio solvent signal (note: CDCl3 referenced at δ 7.27 ppm for ^{1}H NMR and δ 77.16 ppm for ^{13}C NMR, respectively). Data for ^{1}H NMR are reported as follows: chemical shift (δ ppm), multiplicity (s = singlet, d = doublet, t = triplet, q = quartet, m = multiplet, app=apparent, br = broad), coupling constant (Hz), and integration. Data for ^{13}C NMR are reported in terms of chemical shift and no special nomenclature is used for equivalent carbons. High-resolution mass spectrometry data were recorded on a Thermo Q-Exactive instrument using direct injection of samples in dichloromethane into the electrospray source (ESI) with positive ionization. Liquid Chromatography (UPLC) was carried out using a Shimadzu LC-2040C 3D instrument equipped with Chiralpak^{R} AD-H column (250 x 4.6 mm), using an eluent consist of hexane (92%) and isopropanol (8%) at a 1 mL/min flow rate.

Synthesis of fraxinellone and analogs

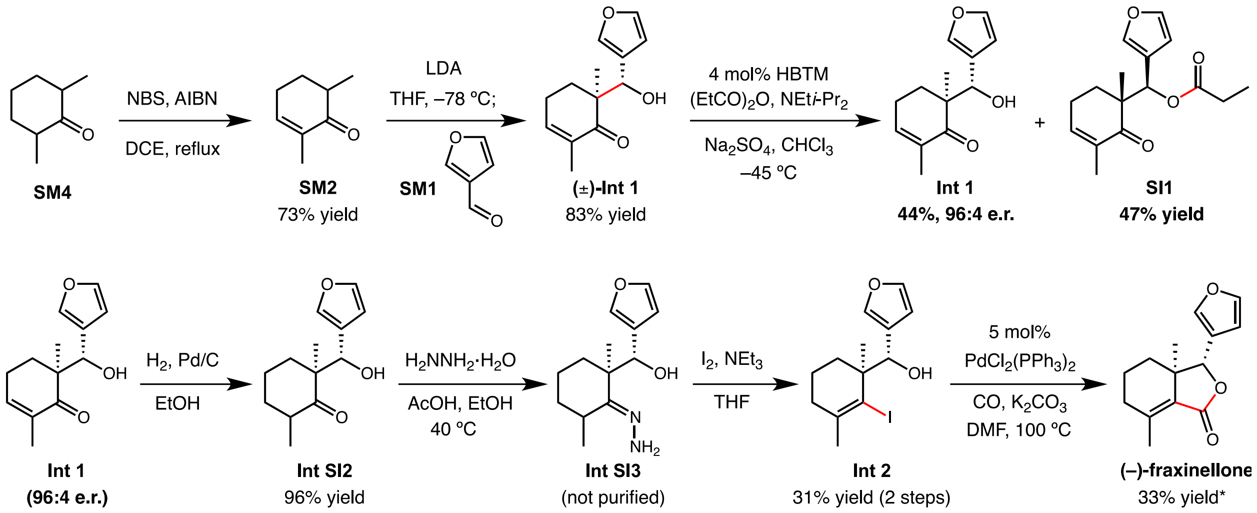

Scheme S1: Enantioselective synthesis of (–)-fraxinellone

2,6-Dimethylcyclohexenone (SM2). Enone **SM2** was synthesized according to a modified literature procedure from Banwell.[1] To a 2-neck round-bottom flask equipped with a magnetic stir bar and a condenser, N-bromosuccinimide (3.15 g, 17.7 mmol) was added to a solution of 2,6-dimethylcyclohexanone **SM4** (2.03 g, 13 mmol) and azobisisobtyronitrile (AIBN) (31.7 mg, 0.193 mmol) in anhydrous 1,2-dichloroethane (13 mL), and the mixture was heated to 90 °C for 24 h. The mixture was then allowed to reach 25 °C, and a sodium hydroxide solution (1 M, 30 mL) was subsequently added. The layers were separated, and the organic layer was washed with brine (25 mL), dried with sodium sulfate and concentrated under reduced pressure. The residue was purified by column chromatography using silica gel and hexane/ethyl acetate (97:3) as the eluent to afford 2,6-dimethylcyclohexenone **(SM2)** as a colorless liquid (1.45 g, 73%). NMR data are in accordance with literature values: ^{1}H NMR (500 MHz, $CDCl_3$): δ = 6.68 (m, 1H), 2.46 – 2.26 (m, 3H), 2.07 – 1.98 (m, 1H), 1.77 (s, 3H), 1.75 – 1.67 (m, 1H), 1.14 (d, *J* = 6.8 Hz, 3H).

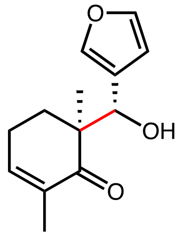

(±)-Int 1

Aldol product (±)-Int 1. Aldol product **(±)-Int 1** was synthesized according to the literature procedure of Fernández-Mateos.[2] In a three-neck round-bottom flask equipped with a magnetic stir bar,

[1] Banwell, M. G.; Jury, J. C. *Org. Prep. Proc. Int.* **2004**, *36*, 87–91.

[2] Fernández-Mateos, A.; Pascual Coca, G.; Pérez Alonso, J. J.; Rubio González, R.; Tapia Hernandéz, C. *Tetrahedron Lett.* **1995**, *36*, 961–964.

diisopropylamine (0.83 g, 1.2 mL, 8.2 mmol) was dissolved in anhydrous tetrahydrofuran (19 mL). The solution was cooled down to 0 °C, and then *n*-BuLi (2.5 M in hexane, 3.3 mL, 8.2 mmol) was added dropwise. The resulting solution was allowed to stir at this temperature for 15 minutes. Then, the reaction mixture was cooled down to -78 °C, and enone **SM2** (0.85 g, 6.8 mmol) was added dropwise. After 30 minutes, freshly distilled 3-furaldehyde (0.78 g, 0.71 mL, 8.2 mmol) was added dropwise at -78 °C and stirred for 45 minutes before quenching the reaction mixture with saturated NH_4Cl solution (19 mL). After stirring at 25 °C for an hour, the reaction mixture was diluted with diethyl ether (15 ml) and the resulting biphasic reaction mixture was then poured into a separatory funnel, and the layers were separated. After extracting the aqueous layer with diethyl ether (3 × 15 mL), the combined organic layers were washed with brine, and dried with sodium sulfate, and concentrated under reduced pressure. The resulting residue was purified by column chromatography using silica gel and hexane/ethyl acetate (93:7) as the eluent to afford racemic aldol product **(±)-Int 1** as a white solid (1.2 g, 83%). NMR data are in accordance with literature values: ^{1}H NMR (500 MHz, $CDCl_3$): δ = 7.38 (s, 1H), 7.36 (t, *J* = 1.7 Hz, 1H), 6.73 (ddt, *J* = 5.8, 2.9, 1.4 Hz, 1H), 6.38 (s, 1H), 4.90 (d, *J* = 1.8 Hz, 1H), 4.52 (d, *J* = 1.8 Hz, 1H), 2.43 – 2.22 (m, 2H), 1.80 (dt, *J* = 2.8, 1.5 Hz, 3H), 1.74 (ddd, *J* = 13.5, 10.8, 5.6 Hz, 1H), 1.51 (m, 1H), 1.19 (s, 3H). ^{13}C NMR (101 MHz, $CDCl_3$): δ = 207.3, 145.7, 142.5, 140.6, 133.9, 124.0, 110.2, 71.6, 47.3, 31.5, 22.6, 16.2, 14.6.

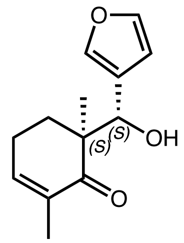

Int 1

Enantioenriched aldol *S,S*-Int 1. Kinetic resolution of racemic aldol **(±)-Int 1** was performed according to a modified literature procedure from Rychnovsky and Newhouse.[3] In an oven-dried two-neck round-bottom flask equipped with a magnetic stir bar, anhydrous sodium sulfate (0.570 g) was flame-dried under high vacuum. After cooling down to room temperature, the reaction vessel was charged with the racemic aldol **(±)-Int 1** (0.285 g, 1.29 mmol) and anhydrous chloroform (2.14 mL) under a nitrogen atmosphere. The resulting mixture was then cooled to -50 °C prior to dropwise addition of a solution (*R*)-Homobenzotetramisole (13.0 mg, 51.6 μmol) in anhydrous chloroform (0.860 mL). After 20 min, *N,N*-diisopropylethylamine (0.183 g, 0.241 mL, 1.42 mmol) and propionic anhydride (0.185 g, 0.183 mL, 1.42 mmol) were added, and the reaction mixture was allowed to stir at -45 °C for 9 h. The reaction was quenched with saturated NH_4Cl solution (3 mL), and was then allowed to reach room temperature. The layers were separated, and the aqueous layer was extracted with chloroform (3 × 4 mL). The combined organic layers were washed with brine, dried with sodium sulfate, and concentrated under reduced pressure. The resulting residue was purified by column chromatography using silica gel and hexane/ethyl acetate (100:0 to 92:8) as the eluent to afford **enantioenriched aldol *S,S*-Int 1.** as a white solid (0.132 g, 46%) and ester ***R,R*-SI1** (0.173 g, 49%). Analysis of **enantioenriched Int 1** by HPLC (hexane

[3] (a) Burns, A. S.; Ross, C. C.; Rychnovsky, S. D. *J. Org. Chem.* **2018**, *83*, 2405–2515. (b) Schuppe, A. W.; Zhao, Y.; Lio, Y.; Newhouse, T. R. *J. Am. Chem. Soc.* **2019**, *141*, 9191–9196.

(92%) and isopropanol (8%) at a 1 mL/min flow rate) indicated a 96:4 enantiomeric ratio (e.r.) of ***S,S*-Int 1** and ***R,R*-Int 1**, respectively.

Enantioenriched aldol Int 1 ^{1}H NMR (500 MHz, $CDCl_3$): δ = 7.38 (dd, *J* = 1.6, 0.8 Hz, 1H), 7.36 (t, *J* = 1.7 Hz, 1H), 6.73 (ddt, *J* = 5.6, 2.9, 1.4 Hz, 1H), 6.38 (d, *J* = 1.8 Hz, 1H), 4.90 (s, 1H), 4.52 (s, 1H), 2.37 (dddd, *J* = 21.0, 10.4, 5.1, 2.5 Hz, 1H), 2.32 – 2.23 (m, 1H), 1.79 (dt, *J* = 2.7, 1.4 Hz, 3H), 1.74 (ddd, *J* = 13.4, 10.8, 5.6 Hz, 1H), 1.51 – 1.47 (m, 1H), 1.19 (s, 3H). ^{13}C NMR (101 MHz, $CDCl_3$): δ = 207.3, 145.7, 142.5, 140.6, 133.9, 124.0, 110.2, 71.6, 47.3, 31.5, 22.6, 16.2, 14.6.

Ester SI1 ^{1}H NMR (400 MHz, $CDCl_3$): δ = 7.32 (t, *J* = 1.7 Hz, 1H), 7.31 (dt, *J* = 1.6, 0.8 Hz, 1H), 6.62 (q, *J* = 3.8 Hz, 1H), 6.38 (s, 1H), 6.30 (dd, *J* = 1.9, 0.9 Hz, 1H), 2.39 – 2.30 (m, 4H), 1.93 – 1.79 (m, 2H), 1.74 (q, *J* = 1.8 Hz, 3H), 1.16 (s, 3H), 1.14 (t, *J* = 7.6 Hz, 4H). ^{13}C NMR (101 MHz, $CDCl_3$) δ = 201.5, 173.3, 143.5, 142.7, 140.8, 134.6, 122.5, 110.3, 71.5, 49.0, 29.8, 27.9, 22.6, 18.8, 16.6, 9.3.

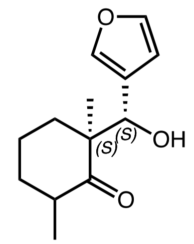

Int SI2

Enantioenriched ketone *(S,S)*-Int SI2

To an 8 mL-glass vial equipped with a magnetic stir bar and a hydrogen balloon, the enantiopure aldol ***S,S*-Int 1** (0.10 g, 0.45 mmol) and palladium on activated charcoal (0.010 g, 10% w/w) were added and the mixture was pumped and back filled with hydrogen (three times). Ethanol (2.4 mL) was then added, and the resulting mixture was stirred at room temperature until TLC showed the full consumption of the starting material (ca. 1 h). The reaction mixture was filtered over a pad of Celite, and was sequentially washed with ethyl acetate (20 mL) and dichloromethane (10 mL). The filtrate was concentrated under reduced pressure to afford ketone ***(S,S)*-Int SI2** as an inconsequential mixture of diastereomers (0.096 mg, 96%) as a white solid. Diastereomer 1: ^{1}H NMR (500 MHz, $CDCl_3$): δ = 7.42 – 7.41 (m, 1H), 7.40 (t, *J* = 1.8 Hz, 1H), 6.39 (t, *J* = 1.3 Hz, 1H), 5.26 (s, 1H), 2.81 (dp, *J* = 12.6, 6.3 Hz, 1H), 2.17 – 2.07 (m, 1H), 1.94 (dddd, *J* = 18.5, 11.9, 8.8, 4.4 Hz, 1H), 1.79 (dtd, *J* = 14.3, 4.3, 2.4 Hz, 1H), 1.71 – 1.63 (m, 2H), 1.48 – 1.35 (m, 3H), 1.09 (d, *J* = 6.5 Hz, 3H), 1.00 (s, 3H). ^{13}C NMR (151 MHz, $CDCl_3$): δ = 216.4, 142.9, 140.2, 124.7, 109.8, 70.1, 53.1, 41.5, 37.1, 35.7, 20.4, 17.0, 15.3. Diastereomer 2: ^{1}H NMR (500 MHz, $CDCl_3$): δ = 7.35 (m, 2H), 6.36 (t, *J* = 1.4 Hz, 1H), 4.84 (s, 1H), 4.01 (s, 1H), 2.73 (dp, *J* = 12.6, 6.3 Hz, 1H), 2.13 – 2.04 (m, 1H), 1.84 (dtq, *J* = 17.4, 10.8, 3.8 Hz, 1H), 1.68 (dp, *J* = 13.9, 3.4 Hz, 1H), 1.52 – 1.49 (m, 2H), 1.34 (qd, *J* = 13.3, 4.1 Hz, 1H), 1.25 (s, 3H), 1.03 (d, *J* = 6.4 Hz, 3H). ^{13}C NMR (151 MHz, $CDCl_3$): δ = 220.9, 142.4, 140.6, 123.9, 110.4, 72.1, 52.3, 41.6, 37.4, 36.5, 20.9, 16.6, 14.7.

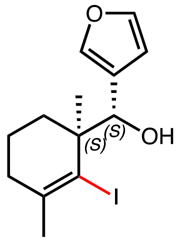

Int 2

Enantioenriched vinyl iodide *S,S*-Int 2

To an ethanolic solution (0.96 mL) of **Int SI2** (mixture of diastereomers) (94 mg, 0.42 mmol), hydrazine monohydrate (0.65 g, 0.63 mL, 13 mmol) and acetic acid (0.22 g, 0.21 mL, 3.7 mmol) were added, and the resulting mixture was heated to 40 °C for 12 h. After cooling to 25 °C, water (4.0 mL) and ethyl acetate (4.0 mL) were added. The layers were separated, and the aqueous layer was extracted with ethyl acetate (2 × 4.0 mL). The combined organic layers were washed with brine, dried with sodium sulfate, quickly passed through a short plug of silica, and concentrated under reduced pressure. The crude hydrazone was azeotropically dried with benzene, and used for the next step without further purification. The resulting hydrazone (**Int SI3)** and anhydrous triethylamine (0.38 g, 0.53 mL, 3.8 mmol) were dissolved in anhydrous tetrahydrofuran (4.3 mL), followed by the dropwise addition of a solution of iodine (0.38 g, 1.5 mmol) in anhydrous tetrahydrofuran (2.7 mL). The addition of iodine was continued until the brown color of the solution remained stable for around 30 sec. The reaction mixture was then diluted with ethyl acetate (10 mL) and washed with saturated sodium thiosulfate solution (6 mL). The aqueous layer was extracted with diethyl ether (3 × 3 mL). The combined organic layers were washed with brine, dried with sodium sulfate, and concentrated under reduced pressure. The resulting residue was purified by column chromatography using silica gel and hexane/ethyl acetate (95:5) to afford the vinyl iodide **Int 2** as an oil (42 mg, 31% over 2 steps). ^{1}H NMR (500 MHz, $CDCl_3$): δ = 7.43 (s, 1H), 7.32 (t, *J* = 1.7 Hz, 1H), 6.44 – 6.42 (m, 1H), 5.02 (s, 1H), 2.09 – 1.94 (m, 2H), 1.87 (d, *J* = 0.9 Hz, 3H), 1.80 (ddd, *J* = 14.0, 10.7, 4.1 Hz, 1H), 1.62 (m, 1H), 1.50 – 1.39 (m, 2H), 1.32 (s, 3H). ^{13}C NMR (101 MHz, $CDCl_3$): δ = 142.4, 141.4, 140.1, 126.5, 113.2, 110.2, 76.0, 46.9, 34.5, 31.5, 29.5, 27.1, 19.2.

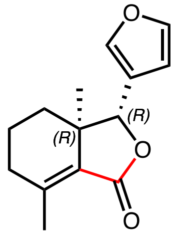

(–)-fraxinellone

Enantioenriched *R,R*-(–)-Fraxinellone

In a Schlenk flask, a mixture of vinyl iodide ***S,S*-Int 2** (42 mg, 0.13 mmol), bis(triphenylphosphine)palladium(II) dichloride (9.1 mg, 13 µmol) and potassium carbonate anhydrous (54 mg, 0.39 mmol) in anhydrous *N,N*-dimethylformamide (0.87 mL) was degassed via three cycles of "freeze-pump-thaw". The reaction vessel was filled with carbon monoxide using a balloon, and was then heated to 100 °C in an oil bath for 16 h. After cooling to room temperature, brine (5 mL) and ethyl acetate (4 mL) were added and the layers were separated. The aqueous layer was then extracted with

ethyl acetate (2 × 4 mL). The combined organic layers were dried with sodium sulfate, and concentrated under reduced pressure. The crude material was purified by preparative thin layer chromatography using silica gel and hexane/ethyl acetate (90:10) to obtain fraxinellone as a white solid (10 mg, 33%). NMR data are in accordance with literature values[4]: ^{1}H NMR (400 MHz, $CDCl_3$): δ = 7.47 (dt, *J* = 1.8, 1.0 Hz, 1H), 7.44 (t, *J* = 1.7 Hz, 1H), 6.35 (dd, *J* = 1.9, 0.9 Hz, 1H), 4.88 (d, *J* = 1.1 Hz, 1H), 2.34 – 2.15 (m, 2H), 2.13 (t, *J* = 0.8 Hz, 3H), 1.90 – 1.66 (m, 3H), 1.50 – 1.39 (m, 1H), 0.86 (d, *J* = 0.7 Hz, 3H).

Analogous reaction to synthesize (±)-fraxinellone. In a 25 mL Schlenk flask, a mixture of vinyl iodide alcohol **(±)-Int 2** (0.250 g, 0.753 mmol), bis(triphenylphosphine)palladium(II) dichloride (26.4 mg, 37.6 μmol) and potassium carbonate anhydrous (312 mg, 2.26 mmol) in anhydrous *N,N*-dimethylformamide (5.0 mL) was degassed via three cycles of "freeze-pump-thaw". The reaction vessel was filled with carbon monoxide using a balloon, and was then heated to 100 °C in an oil bath for 16 h. After cooling to room temperature, brine (50 mL) and ethyl acetate (45 mL) were added and the layers were separated. The aqueous layer was then extracted with ethyl acetate (2 × 45 mL). The combined organic layers were dried with sodium sulfate, and concentrated under reduced pressure. The crude material was purified by column chromatography using silica gel and hexane/ethyl acetate (88:12) to obtain **(±)-fraxinellone** as a white solid (119 mg, 68%).

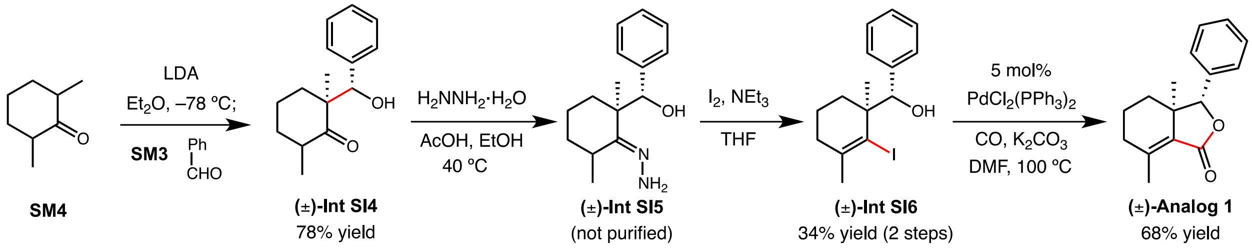

Scheme S2: Synthesis of Analog 1

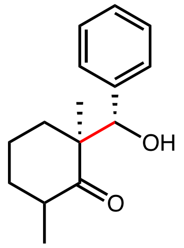

(±)-Int SI4

Aldol product (±)-Int SI4 . Aldol product **(±)-Int SI4** was synthesized according to a modified literature procedure from Fernández-Mateos.[5] In a round-bottom flask equipped with a magnetic stir bar, diisopropylamine (0.672 g, 0.937 mL, 6.64 mmol) was dissolved in anhydrous diethyl ether (12.08 mL).

[4] Guo, Y.; Yan, Y.; Yu, X.; Wang, Y.; Zhi, X.-Y.; Hu, Y.; Xu, H. *J. Agric. Food Chem.* **2012**, *60*, 7016–7021.
[5] Fernández-Mateos, A.; de la Fuente Blanco, J. A. *J. Org. Chem.* **1991**, *56*, 7084–7092.

The solution was cooled down to 0 °C, and then n-BuLi (2.5 M in hexane, 2.66 mL, 6.64 mmol) was added dropwise. The resulting solution was allowed to stir at this temperature for 15 minutes. Then, the reaction mixture was cooled down to -78 °C, and 2,6-dimethylcyclohexanone (0.75 g, 0.811 mL, 6.04 mmol) was added dropwise. After 20 minutes, freshly distilled benzaldehyde (0.64 g, 0.615 mL, 6.04 mmol) was added dropwise at -78 °C and stirred for 1 min before quenching the reaction mixture with saturated NH_4Cl solution (12 mL). After stirring at 25 °C for an hour, the reaction mixture was extracted with diethyl ether (3 x 20 mL), and the combined organic layers were washed with brine, dried with sodium sulfate, and concentrated under reduced pressure. The resulting residue was purified by column chromatography using silica gel and hexane/ethyl acetate (9:1) as the eluent to afford aldol product **Int SI4** (1.097 g, 78%). ^1H NMR (500 MHz, $CDCl_3$): δ = 7.33- 7.29 (m, 4H), 7.29 – 7.27 (m, 1H), 4.92 (s, 1H), 4.21 (s, 1H), 2.80 – 2.71 (m, 1H), 2.11 – 2.05 (m, 1H), 1.83 -1.72 (m, 1H), 1.67 – 1.56 (m, 2H), 1.43 – 1.31 (m, 1H), 1.31 – 1.28 (m, 1H), 1.24 (s, 3H), 1.05 (d, *J* = 6.4 Hz, 3H) . ^{13}C NMR (101 MHz, $CDCl_3$): δ = 221.39, 139.20, 128.39, 127.68, 127.58, 78.20, 52.84, 41.61, 37.99, 36.73, 20.92, 16.05, 14.95. HRMS-ESI calcd for $C_{15}H_{20}O_2Na$ $[M+Na]^+$ 255.1356, found 255.1353.

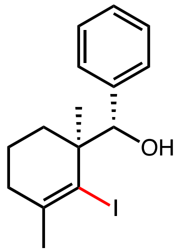

(±)-Int SI6

Hydrazone (±)-Int SI5.

To an ethanolic solution (4.97 mL) of aldol product **Int SI4** (0.52 g, 2.34 mmol), hydrazine monohydrate (3.476 g, 3.37 mL, 69.44 mmol) and acetic acid (1.184 g, 1.11 mL, 19.72 mmol) were added, and the resulting mixture was heated to 42 °C for 3.5 h. After cooling to 25 °C, water (3.0 mL) and ethyl acetate (3.0 mL) were added. The layers were separated, and the aqueous layer was extracted with ethyl acetate (3 x 3.0 mL). The combined organic layers were washed with brine, dried with sodium sulfate, and concentrated under reduced pressure. The crude material was dissolved in ethyl acetate, quickly passed through a short plug of silica, and concentrated under reduced pressure. The crude hydrazine (**Int SI5**) was azeotropically dried with benzene, and used for the next step without further purification (422.7 mg, 73%).

Vinyl iodide (±)-Int SI6.

The resulting hydrazine (**Int SI5**) (422.7 mg, 1.71 mmol) and anhydrous triethylamine (1.696 g, 2.33 mL, 16.758 mmol) were dissolved in anhydrous tetrahydrofuran (19 mL), followed by the dropwise addition of a solution of iodine (1.692 g, 6.669 mmol) in anhydrous tetrahydrofuran (11.9 mL). The addition of iodine was continued until the brown color of the solution remained stable for around 30 sec. The reaction mixture was then diluted with ethyl acetate (5 mL) and washed with saturated sodium

thiosulfate solution (3 mL). The aqueous layer was extracted with ethyl acetate (3 x 5 mL). The combined organic layers were washed with brine, dried with sodium sulfate, and concentrated under reduced pressure. The resulting residue was purified by column chromatography using silica gel and hexane/ethyl acetate (80:20) to afford the vinyl iodide (**Int SI6**) (198 mg, 34%). ^{1}H NMR (500 MHz, $CDCl_3$): δ = 7.54 – 7.46 (m, 2H), 7.32 – 7.19 (m, 3H), 5.13 (s, 1H), 1.99 – 1.90 (m, 2H), 1.85 (s, 3H), 1.77 (dt, *J* = 17.2, 5.7 Hz, 2H), 1.46 (ddd, *J* = 13.7, 10.2, 3.6 Hz, 2H), 1.34 (s, 3H). ^{13}C NMR (125 MHz, $CDCl_3$): δ = 142.19, 141.60, 127.65, 127.26, 126.86, 114.07, 78.80, 47.30, 34.02, 30.43, 29.86, 29.71, 19.14. HRMS-ESI calcd for $C_{15}H_{18}I$ $[M–OH]^+$ 325.0448, found 325.0449.

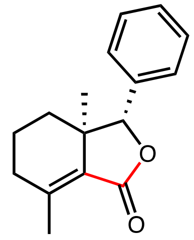

(±)-Analog 1

(±)-Analog 1. In a 25 mL Schlenk flask, a mixture of vinyl iodide **Int SI6** (17 mg, 0.05 mmol), bis(triphenylphosphine)palladium(II) dichloride (3.51 mg, 0.005 mmol) and potassium carbonate anhydrous (23 mg, 0.163 mmol) in anhydrous *N,N*-dimethylformamide (0.36 mL) was degassed via three cycles of "freeze-pump-thaw". The reaction vessel was filled with carbon monoxide using a balloon, and was then heated to 105 °C in an oil bath for 20 h. After cooling to room temperature, brine (5 mL) and ethyl acetate (5 mL) were added and the layers were separated. The aqueous layer was then extracted with ethyl acetate (3 x 5 mL). The combined organic layers were washed with an aqueous solution of LiCl (5%), dried with sodium sulfate, and concentrated under reduced pressure. The crude material was purified by column chromatography using silica gel and hexane/ethyl acetate (95:5) to obtain **Analog 1** (8.30 mg, 68%). ^{1}H NMR (500 MHz, $CDCl_3$): δ = 7.40 – 7.30 (m, 5H), 4.99 (s, 1H), 2.28 (dd, *J* = 19.8, 6.8 Hz, 1H), 2.22 – 2.17 (m, 1H), 2.16 (s, 3H), 1.88 – 1.80 (m, 2H), 1.77 – 1.66 (m, 1H), 1.62 – 1.55 (m, 1H), 0.69 (s, 3H) . ^{13}C NMR (101 MHz, $CDCl_3$): δ = 170.16, 148.58, 135.97, 128.41, 128.04, 127.94, 125.65, 88.53, 43.75, 32.21, 32.10, 20.39, 18.65, 18.33.

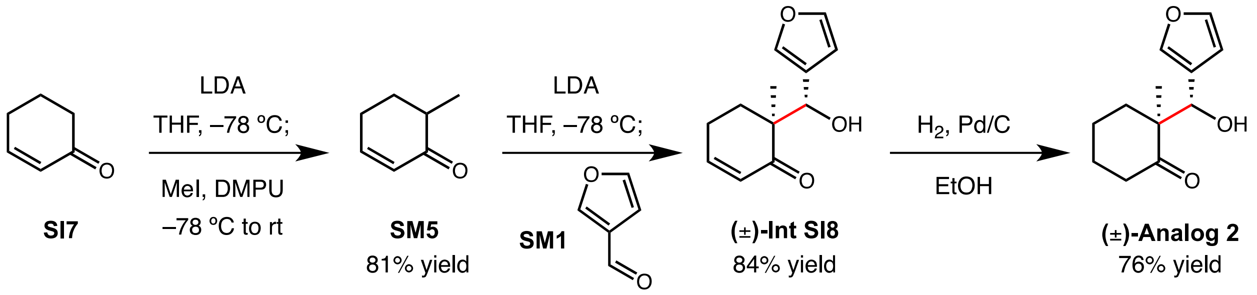

Scheme S3: Synthesis of Analog 2

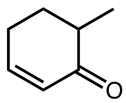

SM5

6-Methylcyclohexenone (SM5). Enone **SM5** was synthesized according to a modified literature procedure.[6] In a three-neck round-bottom flask equipped with a magnetic stir bar and an addition funnel, diisopropylamine (6.91 g, 9.60 mL, 68.3 mmol) was dissolved in anhydrous tetrahydrofuran (36 mL). The solution was cooled down to 0 °C, and then using the addition funnel, *n*-BuLi (2.5 M in hexane, 25.5 mL, 62.5 mmol) was added dropwise. The resulting solution was allowed to stir at this temperature for 30 minutes. Then, the reaction mixture was cooled down to -78 °C, and a solution of cyclohexenone (3.46 g, 3.5 mL, 36.0 mmol) in THF (45 mL) was added dropwise. After 30 minutes, iodomethane (13.0 g, 5.7 mL, 91.6 mmol) was added dropwise at -78 °C and stirred for 30 minutes. *N,N*-dimethylpropyleneurea (27 mL) was then added, and the reaction mixture was allowed to gradually reach the room temperature. After 12 h, saturated NH_4Cl solution (30 mL) was slowly added at 0 °C, and the reaction mixture was extracted with diethyl ether (2 x 35 ml). The combined organic layers were washed with brine, and dried with sodium sulfate, and concentrated under reduced pressure. The resulting residue was purified by column chromatography using silica gel and hexane/ethyl ether (97:3) as the eluent to afford 6-methylcyclohexenone **SM5** as a colorless liquid (3.22 g, 81%). NMR data are in accordance with literature values: ^{1}H NMR (500 MHz, $CDCl_3$): δ = 6.96 – 6.88 (m, 1H), 5.99 (dt, *J* = 10.1, 2.0 Hz, 1H), 2.46 – 2.33 (m, 3H), 2.11 – 2.03 (m, 1H), 1.74 (dddd, *J* = 13.4, 11.9, 8.4, 6.7 Hz, 1H), 1.15 (d, *J* = 6.8 Hz, 3H). HRMS-ESI calcd for $C_7H_{11}O$ $[M+H]^+$ 111.0804, found 111.0804.

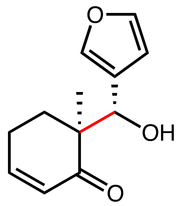

(±)-Int SI8

Aldol product (±)-Int SI8. In a three-neck round-bottom flask equipped with a magnetic stir bar, diisopropylamine (0.59 g, 0.82 mL, 5.8 mmol) was dissolved in anhydrous tetrahydrofuran (19 mL). The solution was cooled down to 0 °C, and then *n*-BuLi (2.5 M in hexane, 2.3mL, 5.8 mmol) was added dropwise. The resulting solution was allowed to stir at this temperature for 15 minutes. Then, the reaction mixture was cooled down to -78 °C, and enone **SM5** (0.50 g, 4.5 mmol) was added dropwise. After 30 minutes, freshly distilled 3-furaldehyde (0.56 g, 0.51 mL, 5.8 mmol) was added dropwise at -78 °C and stirred for 45 minutes before quenching the reaction mixture with saturated NH_4Cl solution

---

[6] Marques, F. A.; Lenz, C. A.; Simonelli, F.; Sales Maia, B. H. L. N.; Vellasco, A. P.; Eberlin, M. N. *J. Nat. Prod.* **2004**, *67*, 1939–1941.

(25 mL). After stirring at 25 °C for an hour, the reaction mixture was diluted with diethyl ether (15 ml) and the layers were separated. After extracting the aqueous layer with diethyl ether (3 × 10 mL), the combined organic layers were washed with brine, and dried with sodium sulfate, and concentrated under reduced pressure. The resulting residue was purified by column chromatography using silica gel and hexane/ethyl acetate (85:15) as the eluent to afford aldol **Int SI8** as a white solid (0.78 g, 84%). ^1H NMR (400 MHz, $CDCl_3$): δ = 7.38 (dt, *J* = 1.5, 0.8 Hz, 1H), 7.37 (t, *J* = 1.7 Hz, 1H), 7.00 – 6.92 (m, 1H), 6.38 (dd, *J* = 1.9, 0.9 Hz, 1H), 5.96 (ddd, *J* = 10.0, 2.5, 1.5 Hz, 1H), 4.91 (s, 1H), 4.40 (s, 1H), 2.46 – 2.28 (m, 2H), 1.74 (ddd, *J* = 13.5, 10.0, 6.2 Hz, 1H), 1.55 (dddd, *J* = 13.6, 4.7, 3.3, 1.3 Hz, 1H), 1.20 (s, 3H). ^{13}C NMR (101 MHz, $CDCl_3$): δ = 207.1, 150.6, 142.7, 140.8, 128.4, 124.0, 110.3, 71.6, 47.7, 31.1, 23.1, 14.7. HRMS-ESI calcd for $C_{12}H_{14}O_3Na$ $[M+Na]^+$ 229.0835, found 229.0832.

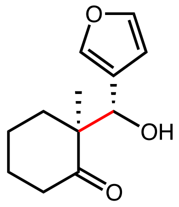

(±)-Analog 2

To an 20 mL-glass vial equipped with a magnetic stir bar, aldol product **Int SI8** (0.277 g, 1.342 mmol) and palladium on activated charcoal (27.7 mg, 10% w/w) were added and the mixture was first flushed with nitrogen gas. Ethanol (6.71 mL) was then added, and the mixture was flushed with hydrogen gas using a balloon. The resulting mixture was stirred at room temperature until TLC showed the full consumption of the starting material (ca. 2 h). The reaction mixture was filtered over a pad of Celite, and was sequentially washed with diethyl ether and dichloromethane. The filtrate was concentrated under reduced pressure, and the resulting residue was purified by column chromatography using silica gel and hexane/diethyl ether (7:3) as the eluent to afford **Analog 2** (212.7 mg, 76%) as a white solid (upon standing in freezer overnight). ^1H NMR (600 MHz, $CDCl_3$): δ = 7.37 (s, 1H), 7.36 (s, 1H), 6.37 (dd, *J* =1.4 Hz, 1H), 4.90 (s, 1H), 3.86 (s, 1H), 2.63 – 2.55 (m, 1H), 2.38 – 2.32 (m, 1H), 2.07 – 2.00 (m, 1H), 1.77 – 1.64 (m, 3H), 1.53 – 1.48 (m, 2H), 1.22 (s, 3H). ^{13}C NMR (151 MHz, $CDCl_3$): δ = 219.43, 142.60, 140.66, 123.89, 110.39, 71.77, 52.39, 39.16, 36.72, 27.37, 20.79, 16.48. HRMS-ESI calcd for $C_{12}H_{16}O_3Na$ $[M+Na]^+$ 231.0992, found 231.0990.

NMR Spectra

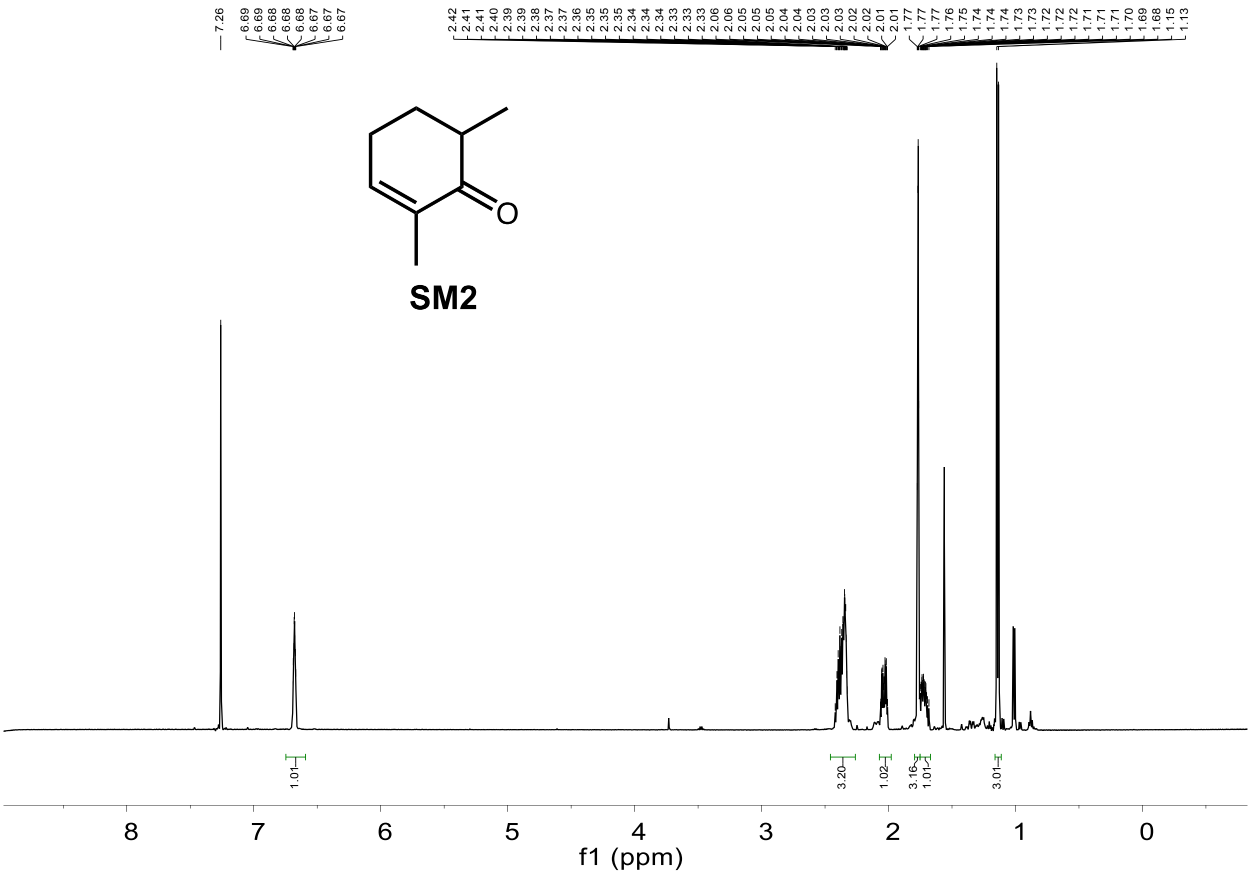

Figure S4: ^{1}H NMR spectra of **SM2** in $CDCl_3$

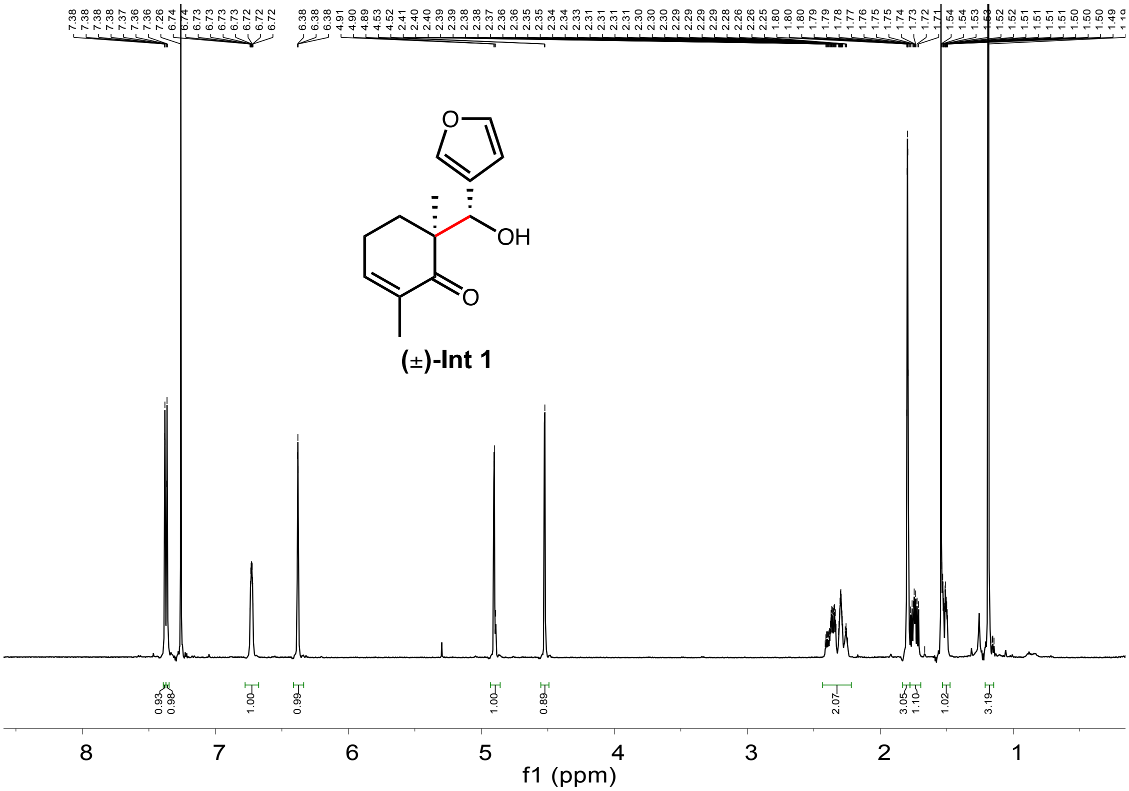

Figure S5: ^{1}H NMR spectra of **(±)-Int 1** in $CDCl_3$

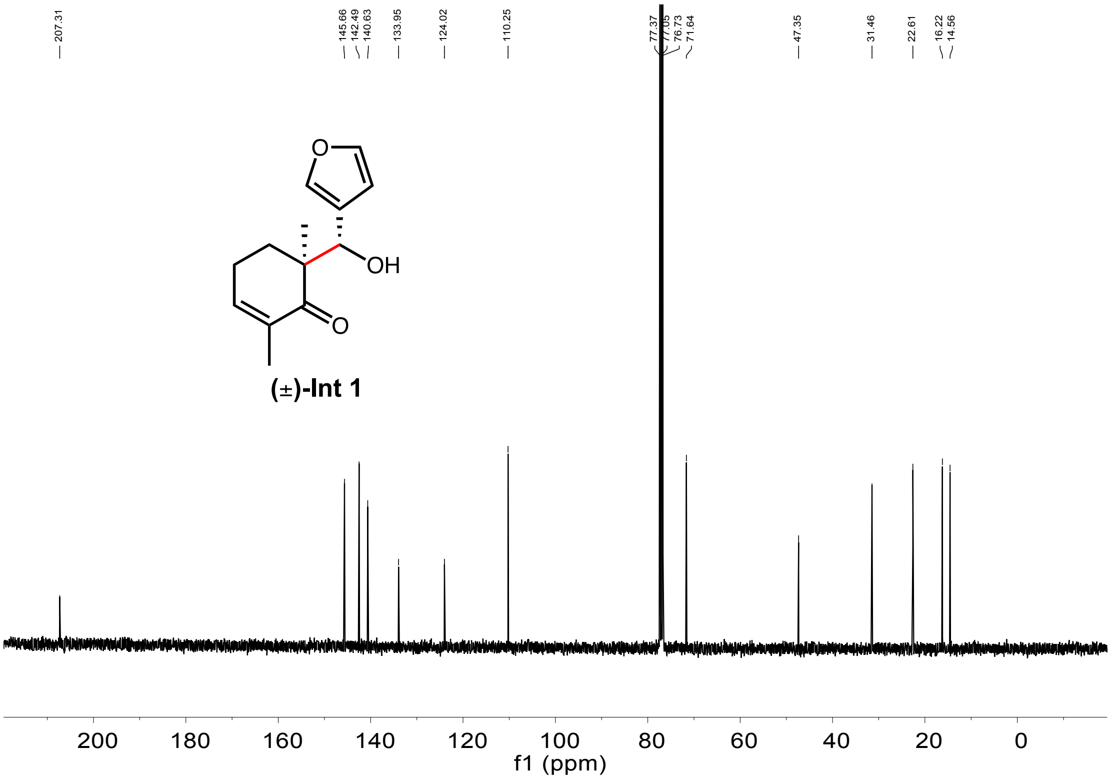

Figure S6: ^{13}C NMR spectra of **(±)-Int 1** in $CDCl_3$

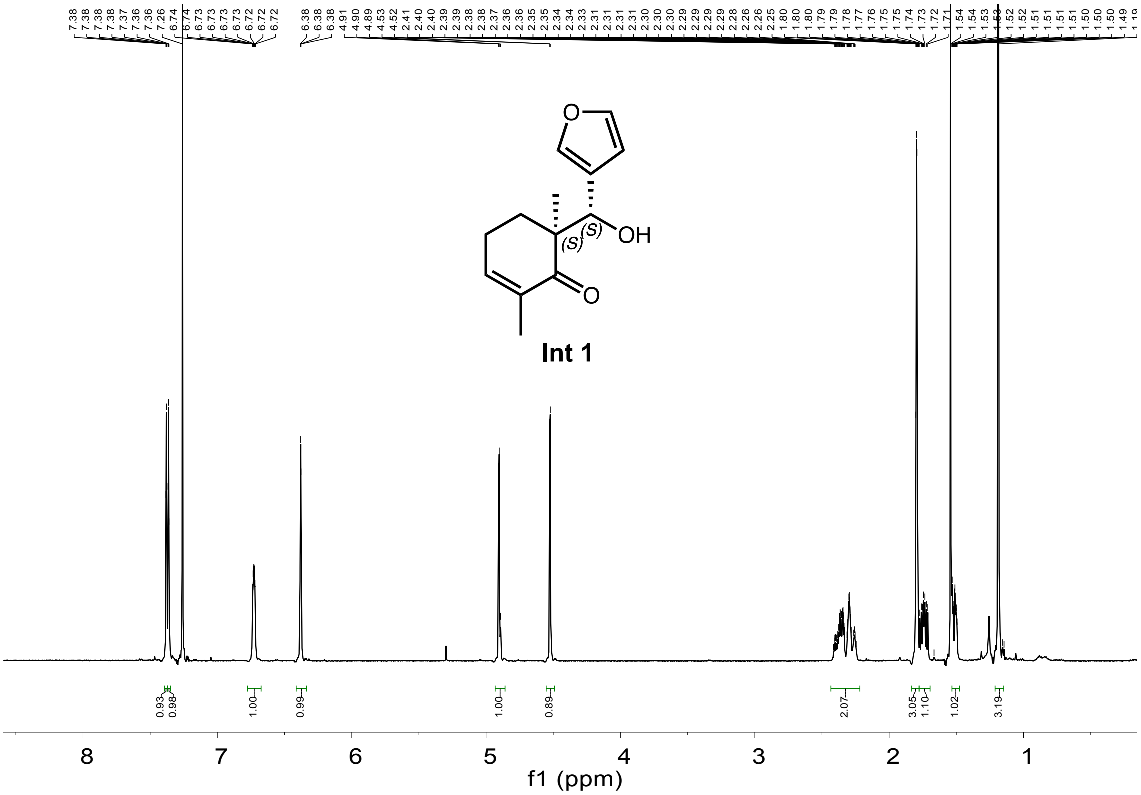

Figure S7: ^{1}H NMR spectra of **Int 1** in $CDCl_3$

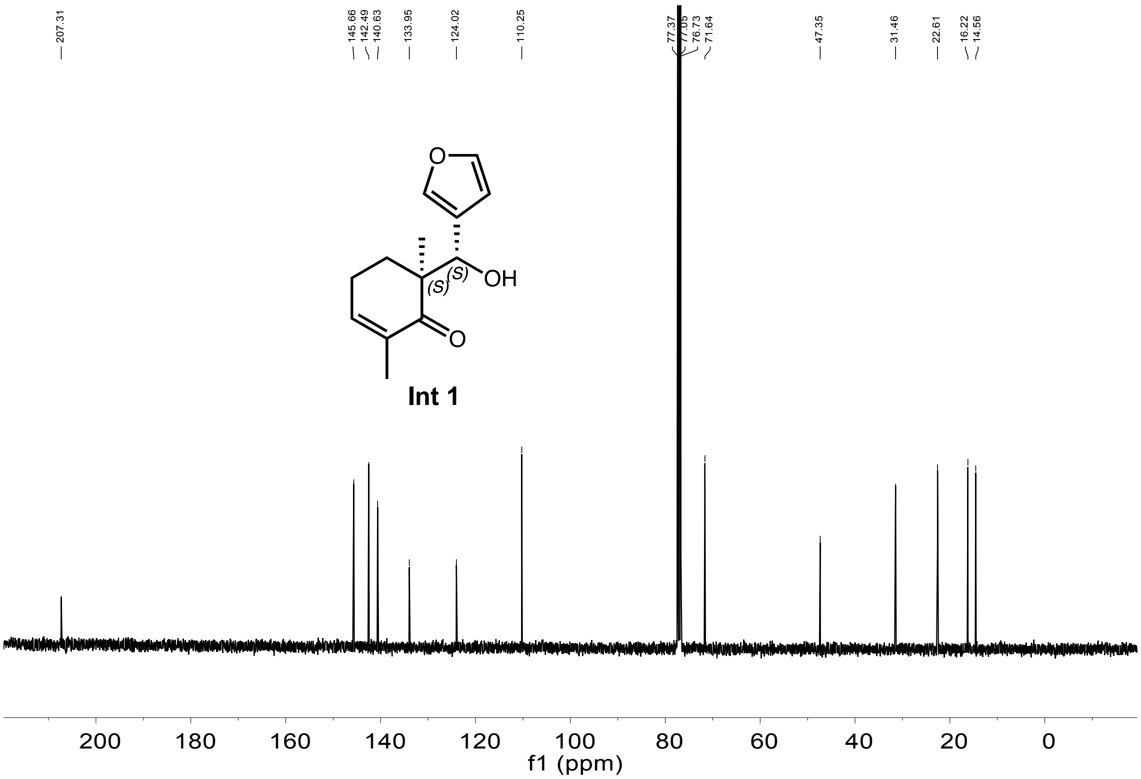

Figure S8: ^{13}C NMR spectra of **Int 1** in $CDCl_3$

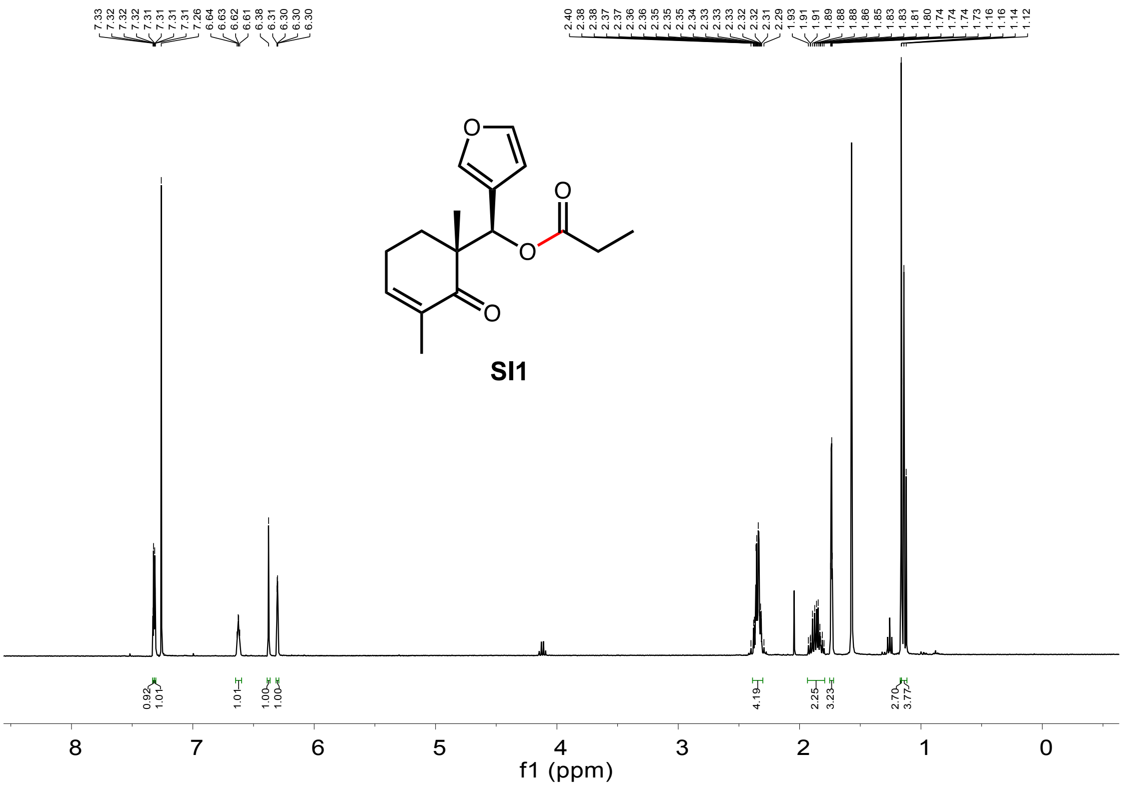

Figure S9: ^{1}H NMR spectra of Ester **SI1** in $CDCl_3$

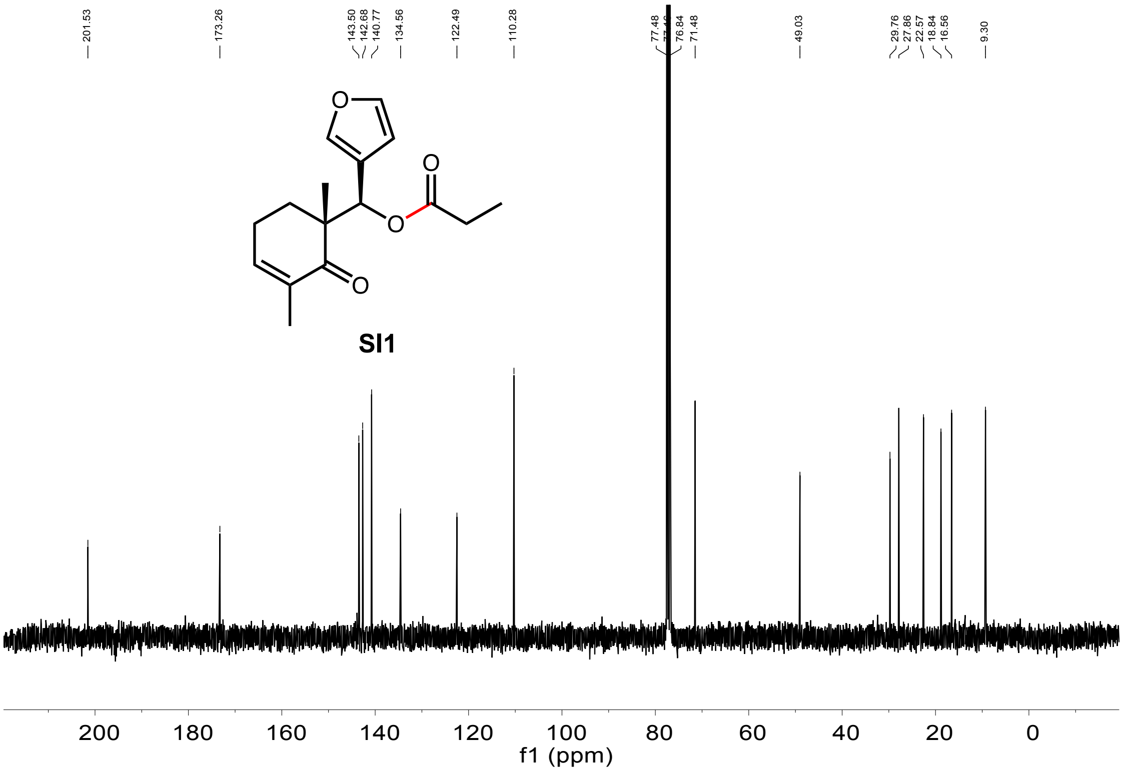

Figure S10: ^{13}C NMR spectra of Ester **SI1** in $CDCl_3$

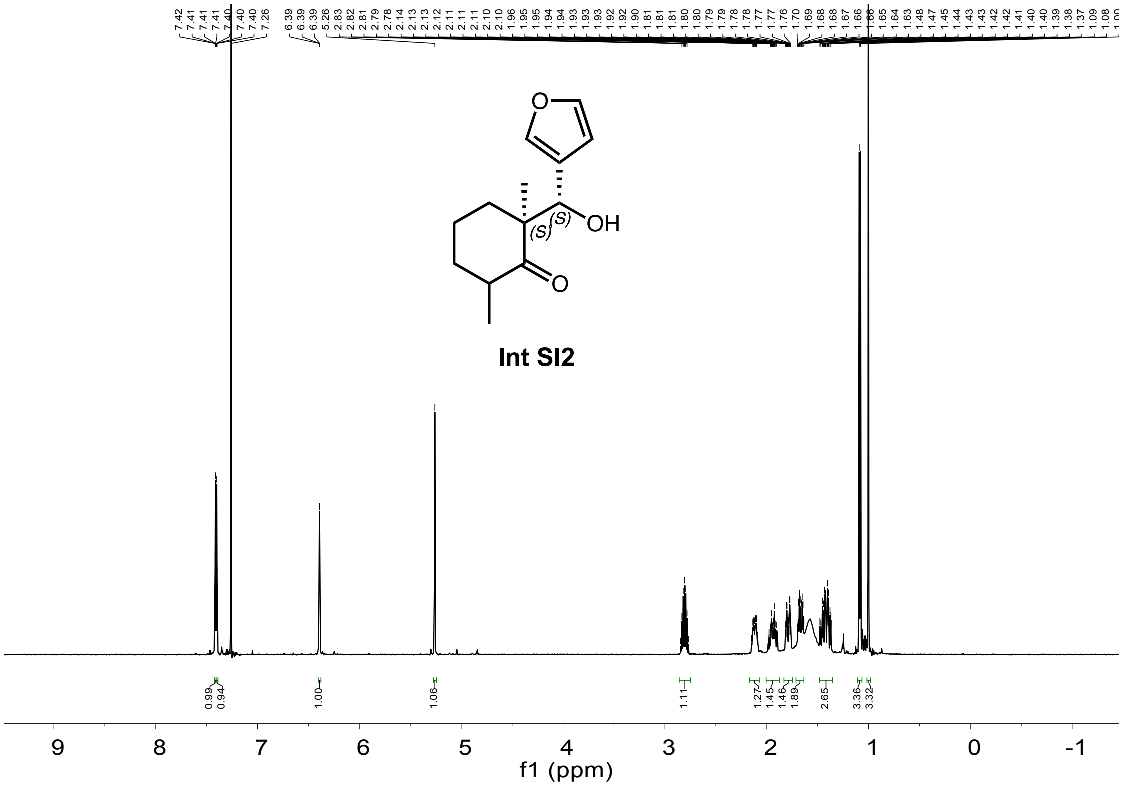

Figure S11: ^{1}H NMR spectra of **Int SI2** in $CDCl_3$

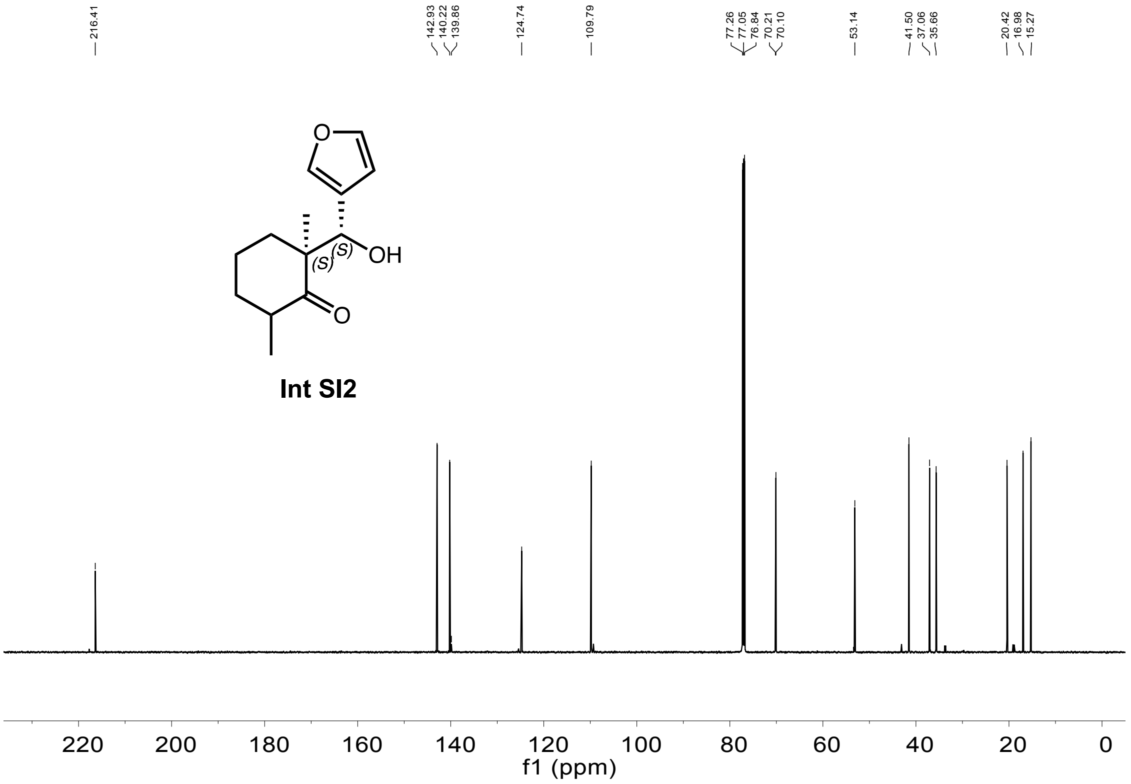

Figure S12: ^{13}C NMR spectra of **Int SI2** in $CDCl_3$

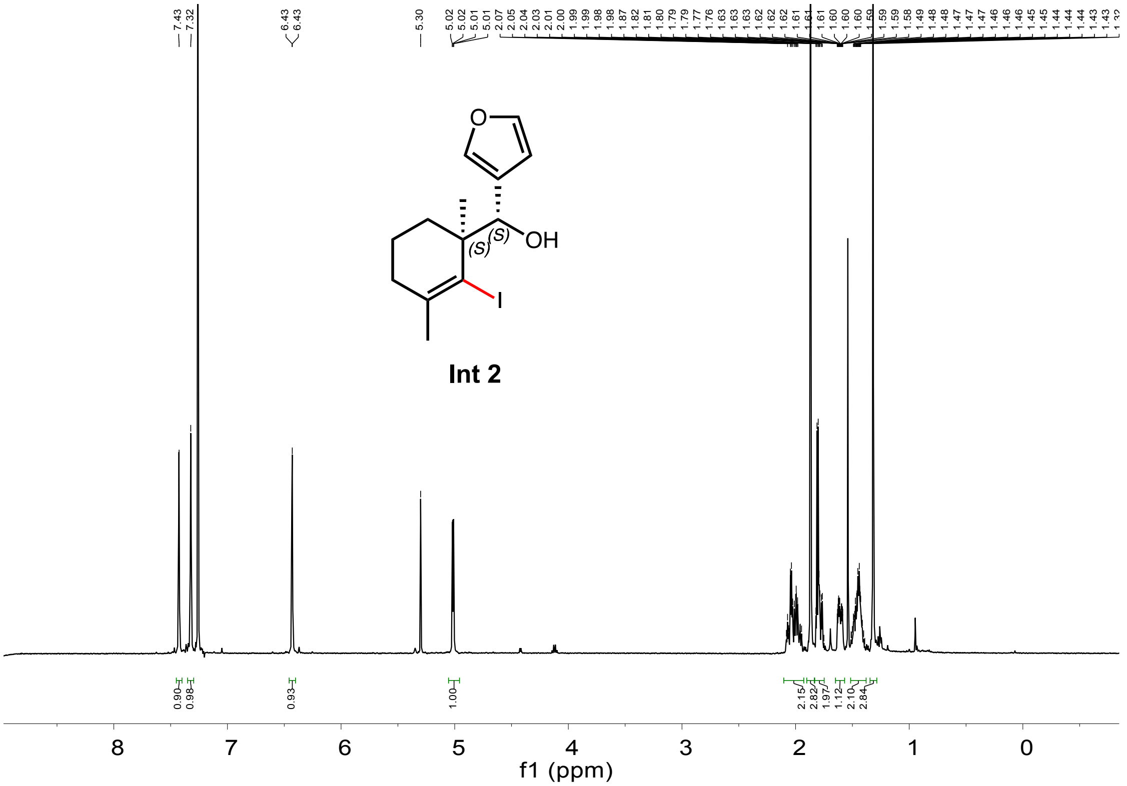

Figure S13: ^{1}H NMR spectra of **Int 2** in $CDCl_3$

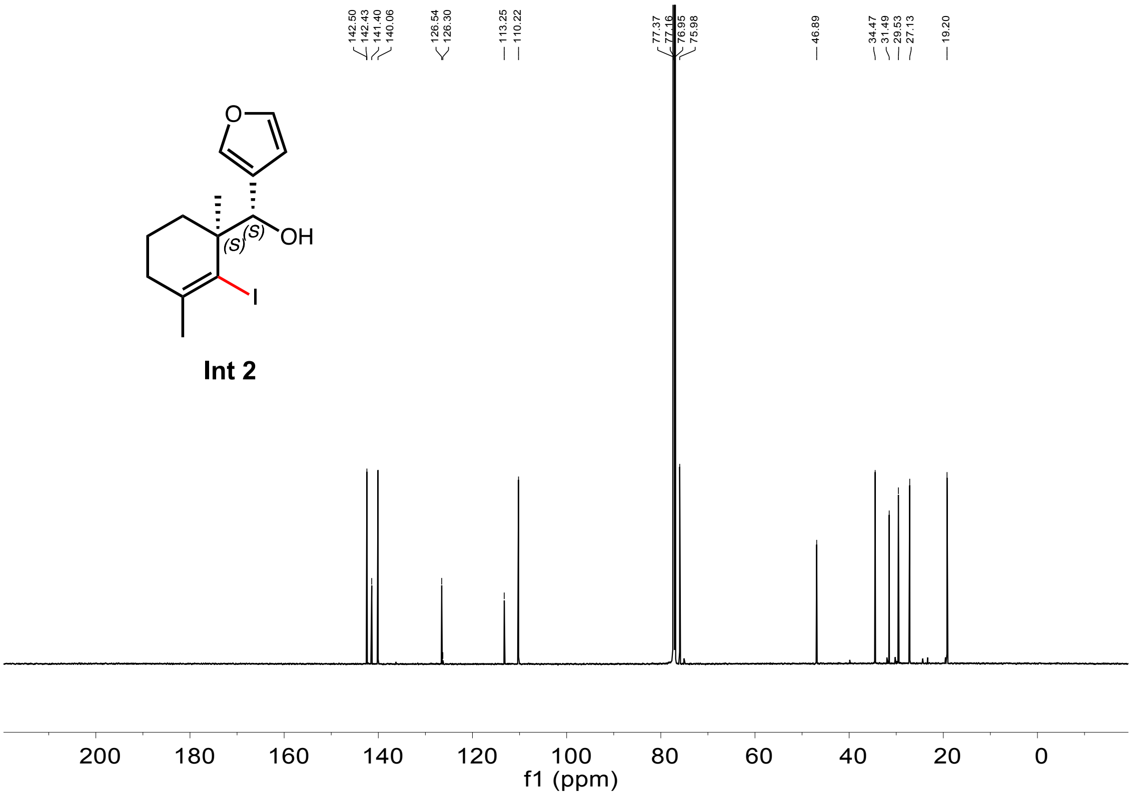

Figure S14: ^{13}C NMR spectra of **Int 2** in $CDCl_3$

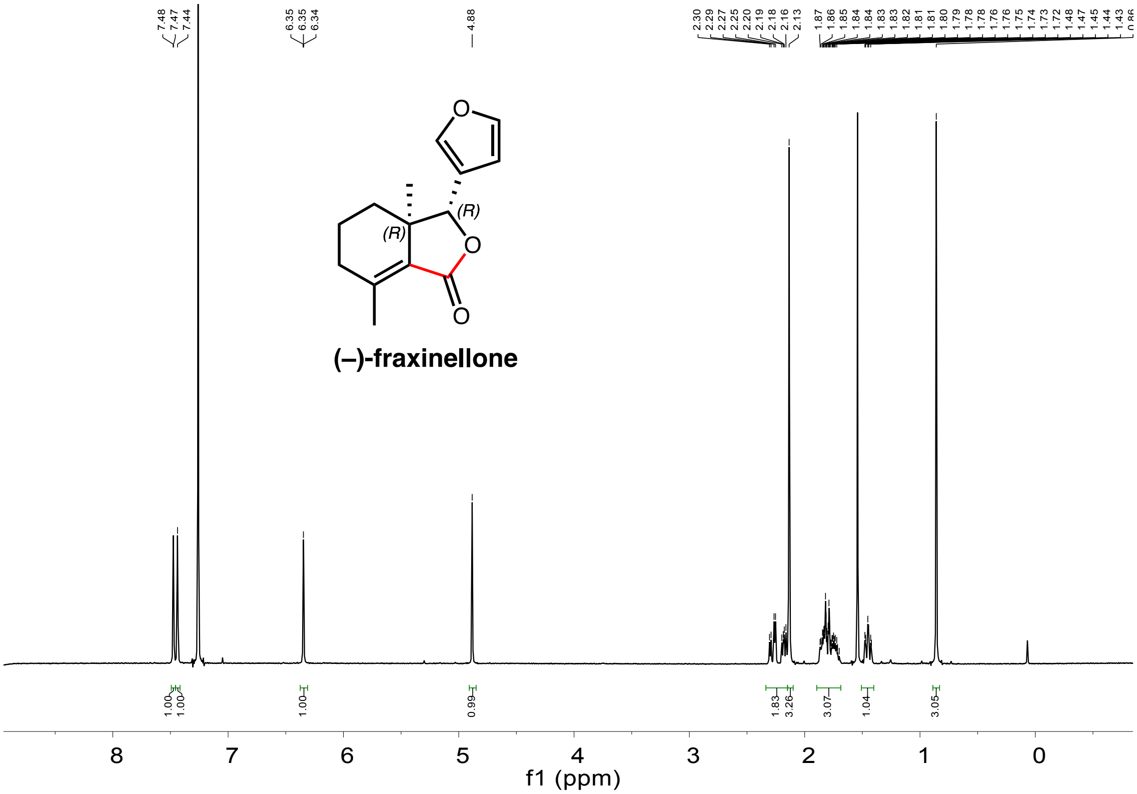

Figure S15: ^{1}H NMR spectra of Enantioenriched ***R,R*-(–)-Fraxinellone** in $CDCl_3$

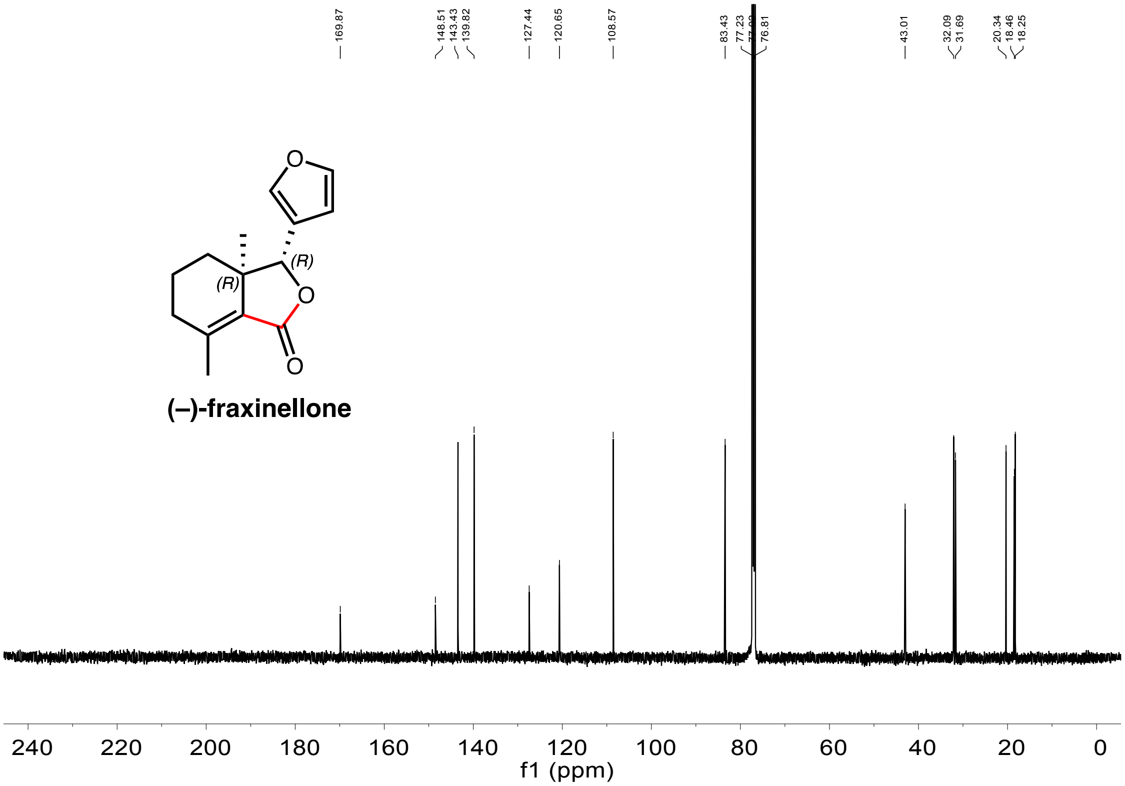

Figure S16: ^{13}C NMR spectra of Enantioenriched ***R,R*-(–)-fraxinellone** in $CDCl_3$

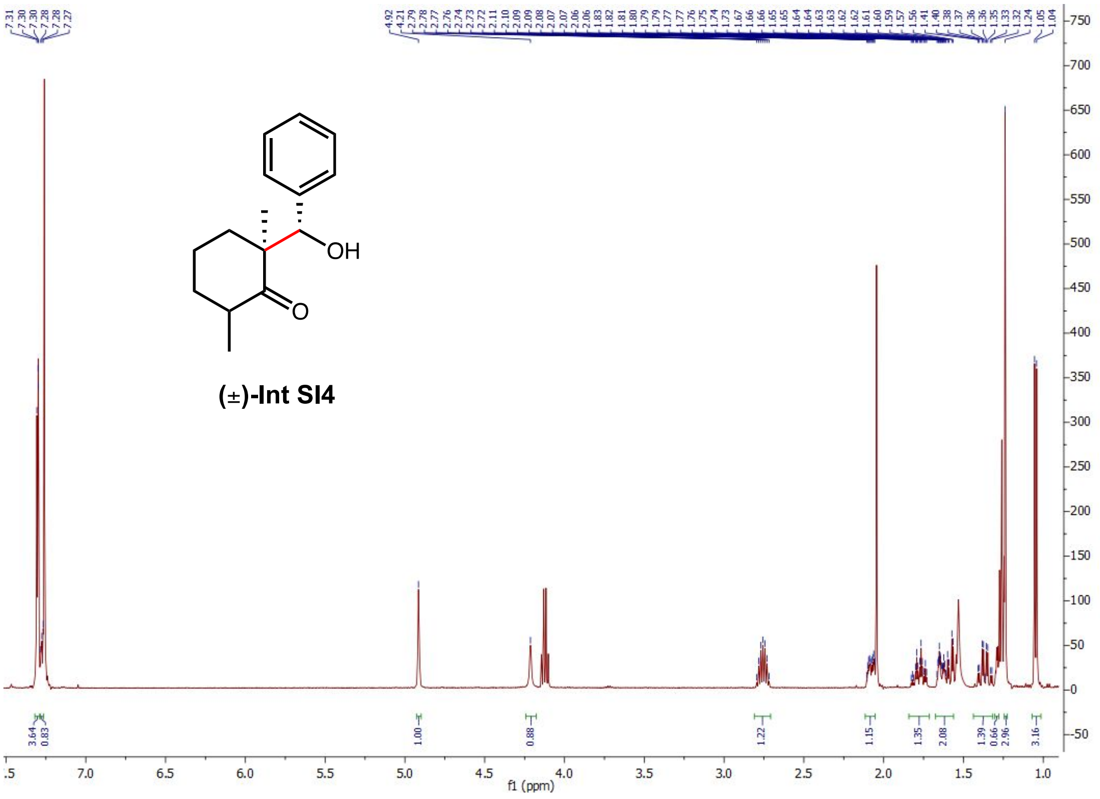

Figure S17: ^{1}H NMR spectra of **Int SI4** in $CDCl_3$

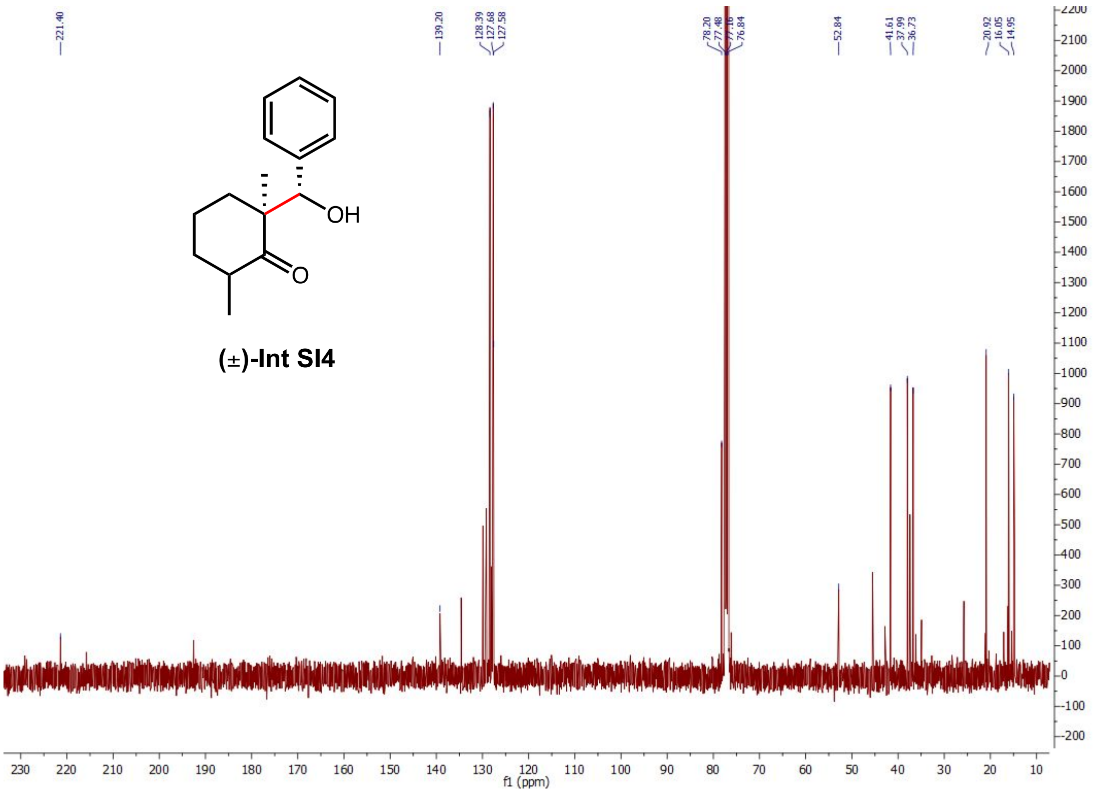

Figure S18: ^{13}C NMR spectra of **Int SI4** in $CDCl_3$

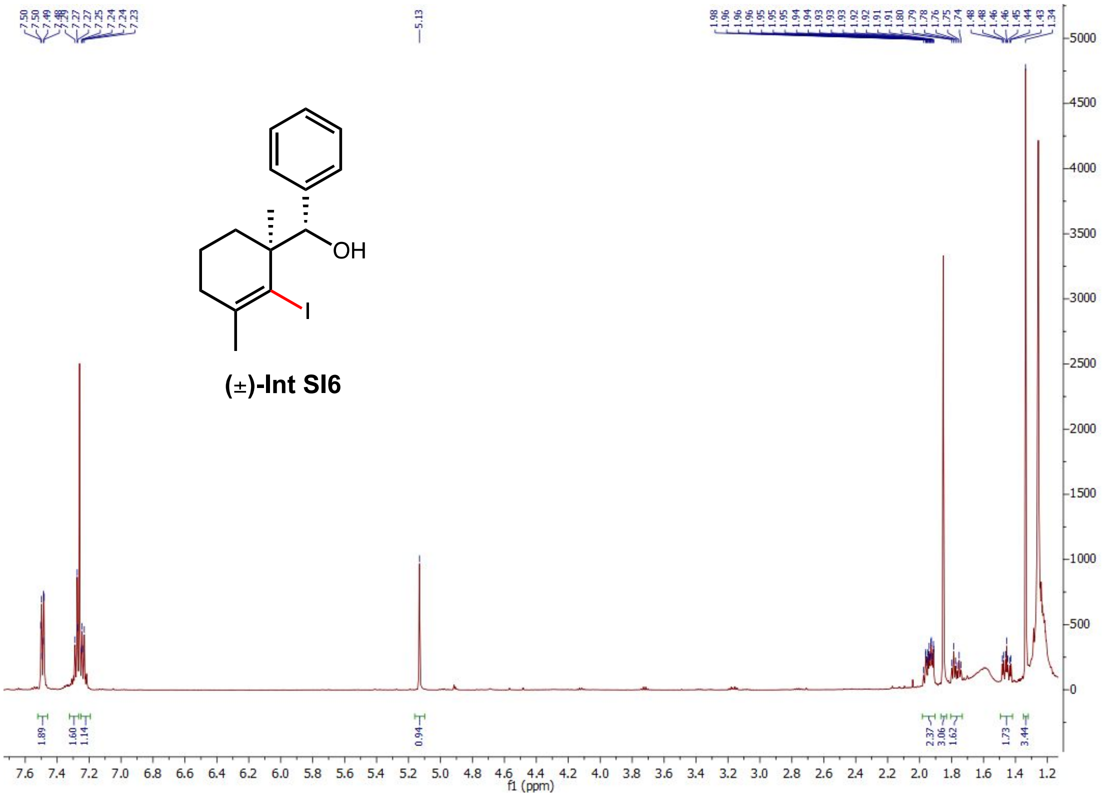

Figure S19: ^{1}H NMR spectra of **Int SI6** in $CDCl_3$

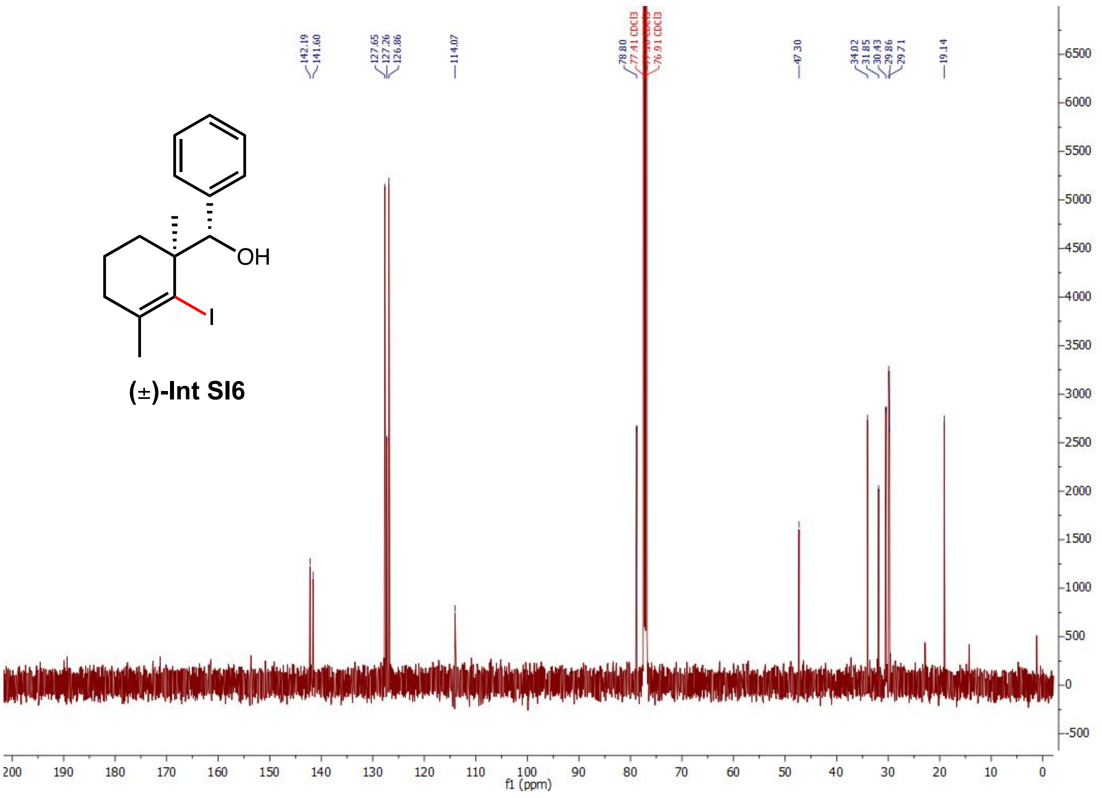

Figure S20: ^{13}C NMR spectra of **Int SI6** in $CDCl_3$

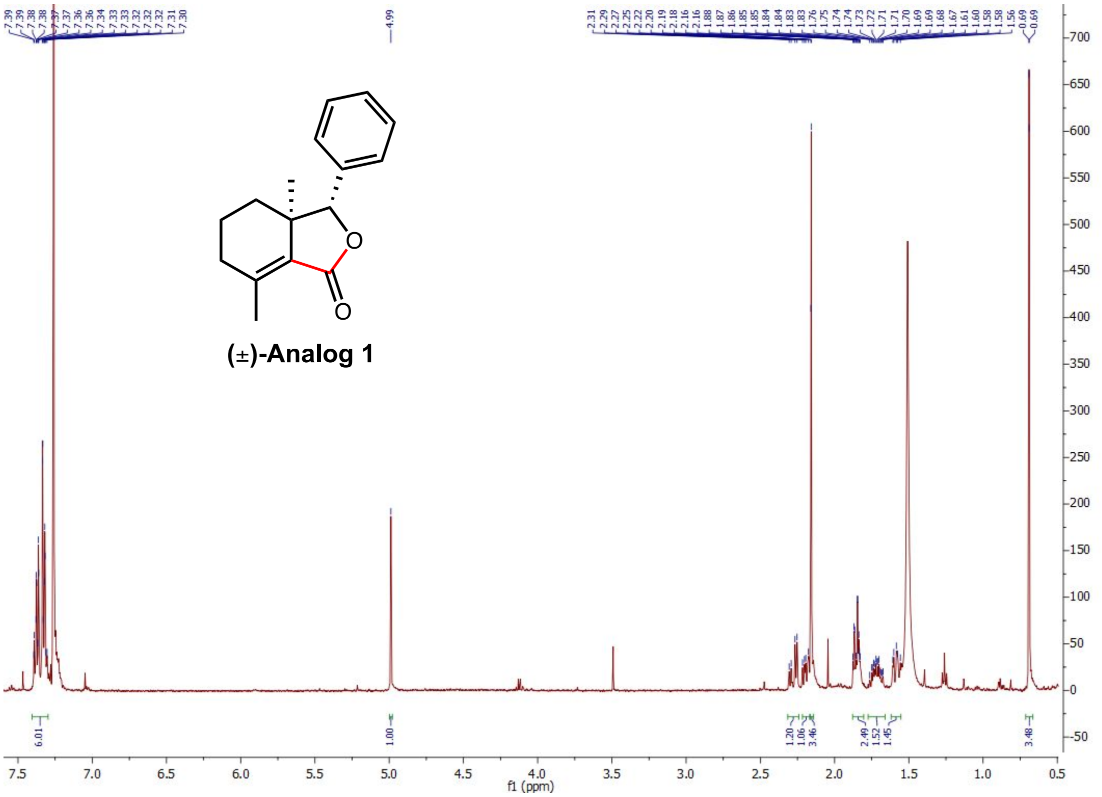

Figure S21: ^{1}H NMR spectra of **Analog 1** in $CDCl_3$

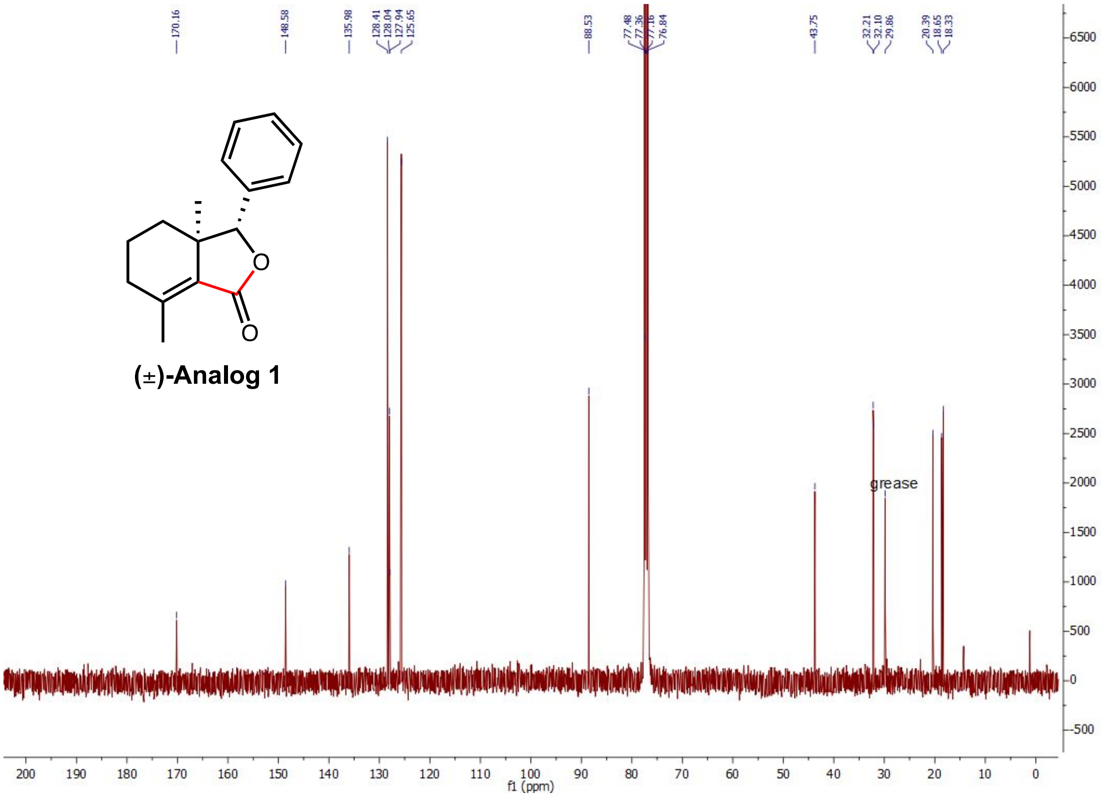

Figure S22: ^{13}C NMR spectra of **Analog 1** in $CDCl_3$

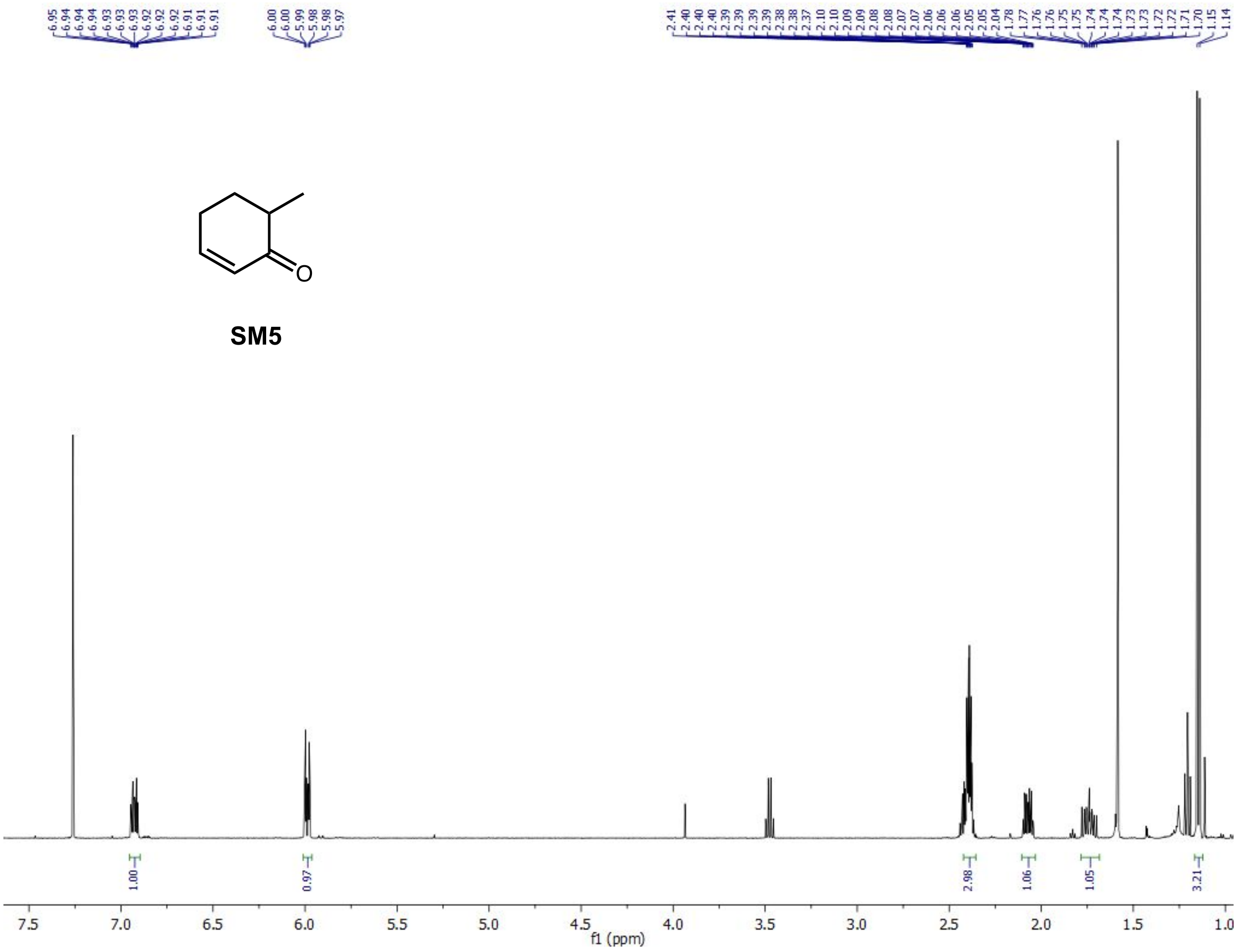

Figure S23: ^{1}H NMR spectra of **SM5** in $CDCl_3$

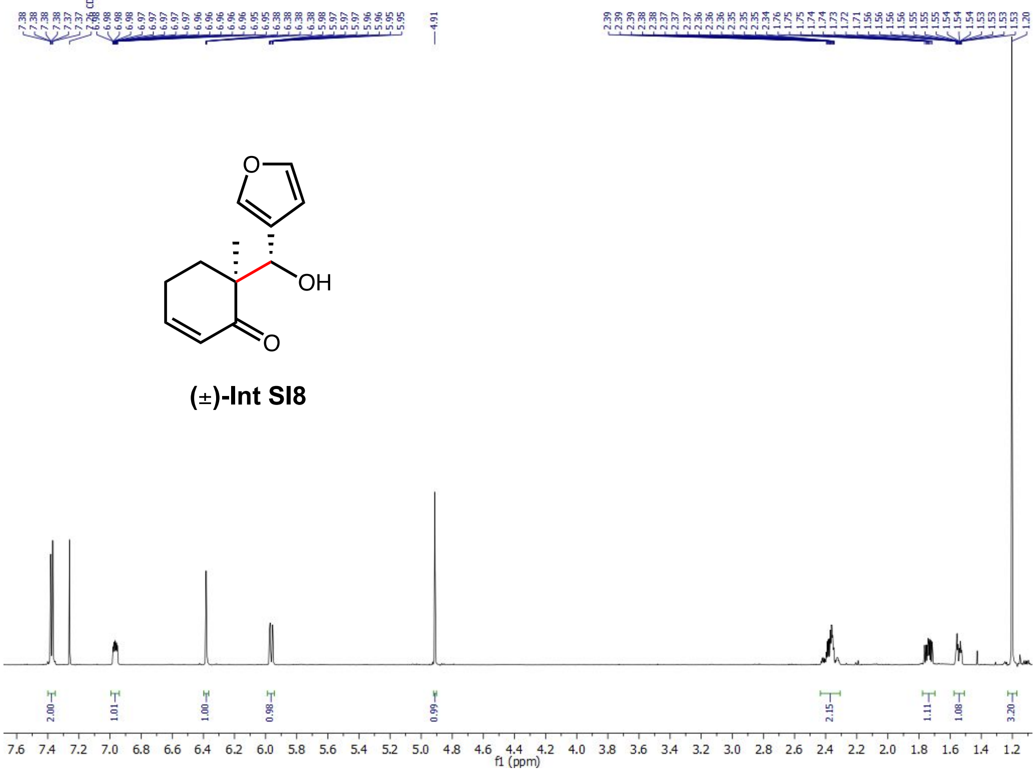

Figure S24: ^{1}H NMR spectra of **Int SI8** in $CDCl_3$

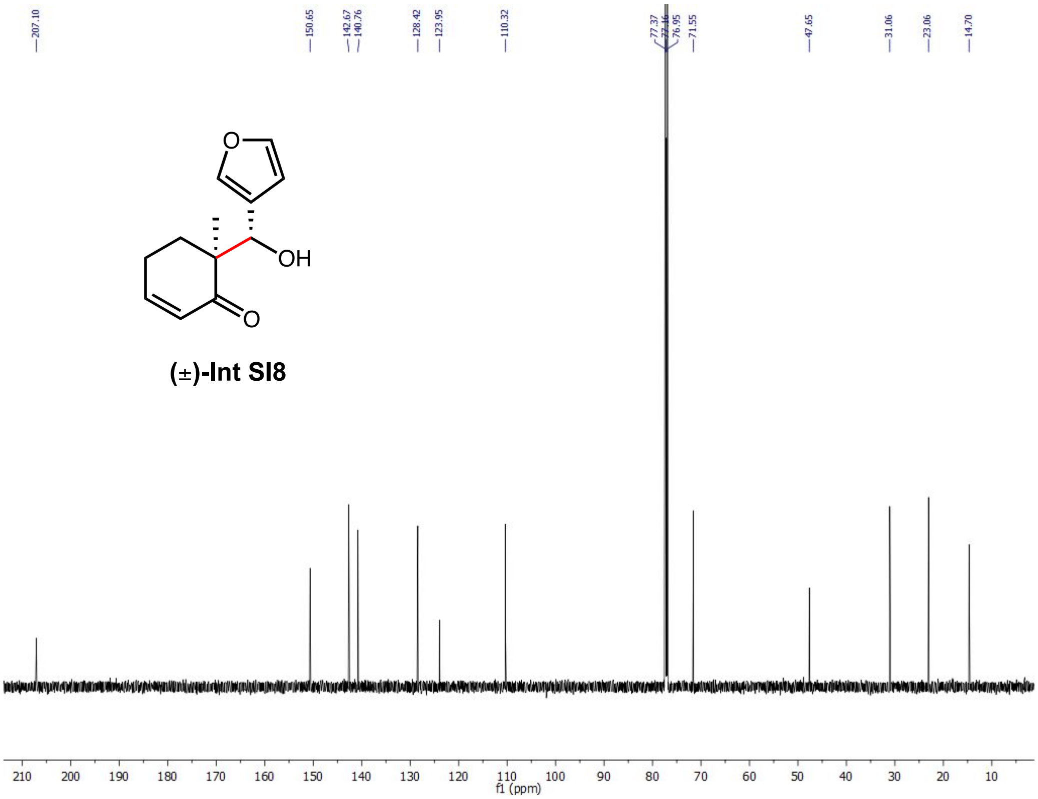

Figure S25: ^{13}C NMR spectra of **Int SI8** in $CDCl_3$

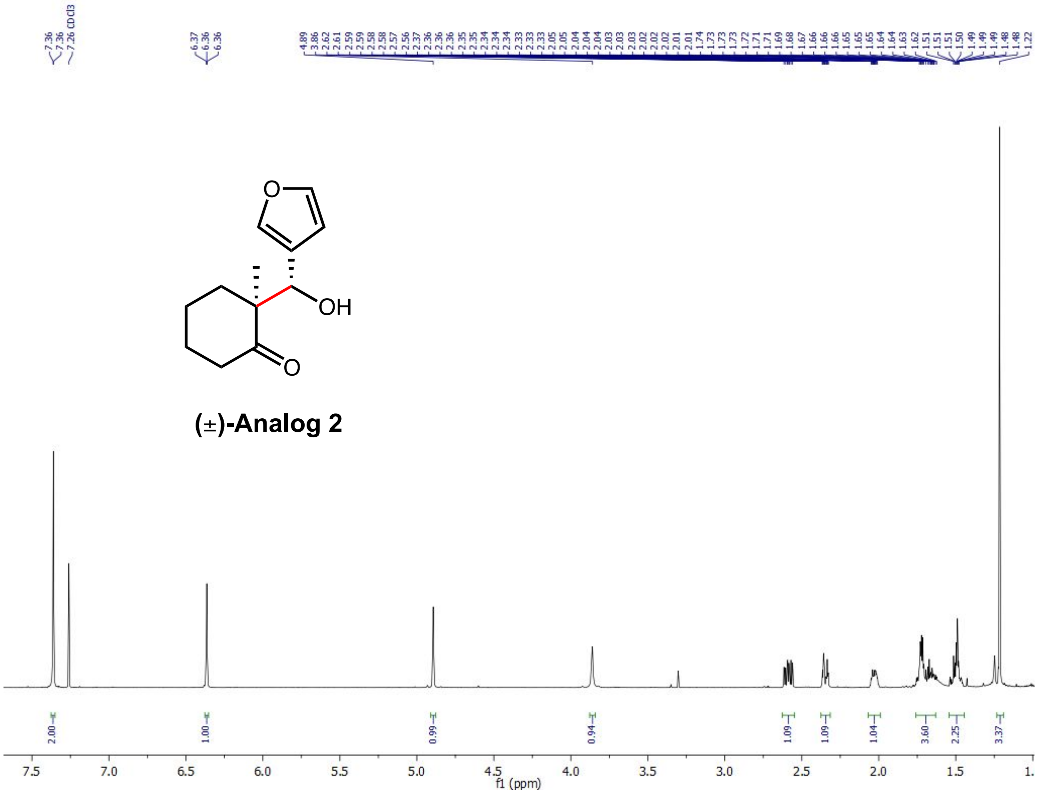

Figure S26: ^{1}H NMR spectra of **Analog 2** in $CDCl_3$

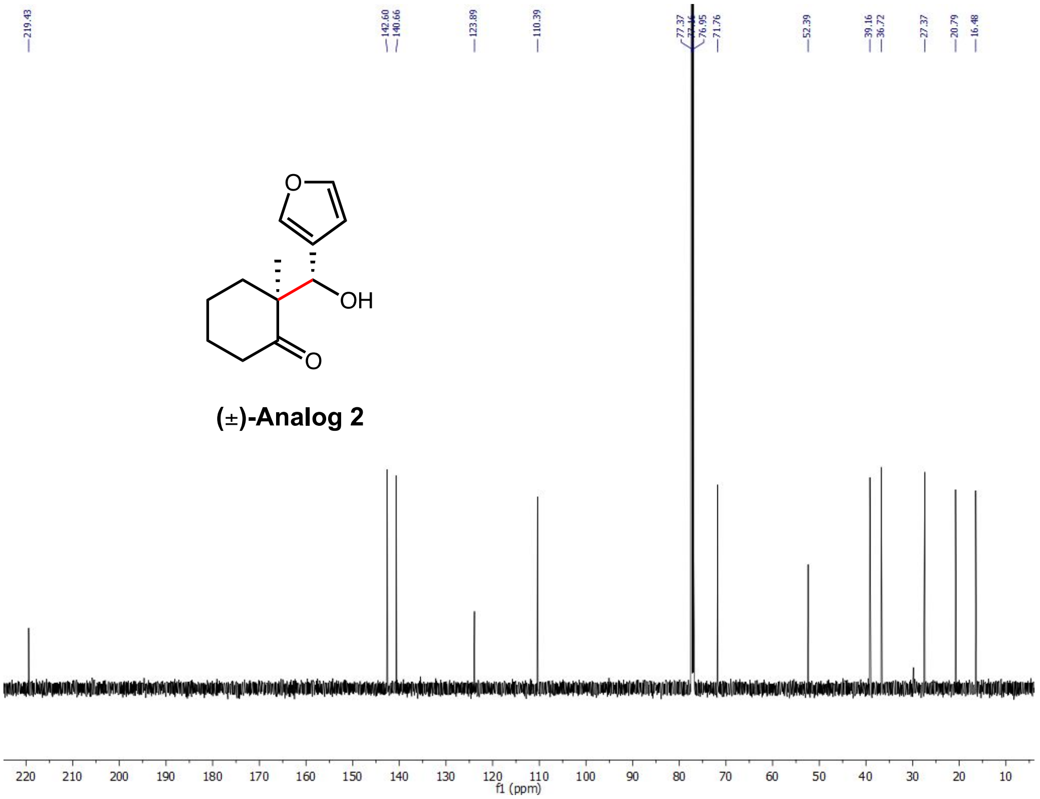

Figure S27: ^{13}C NMR spectra of **Analog 2** in $CDCl_3$
